# Bioinspired honeycomb-mimetic adaptive hyaluronic acid hydrogel composite scaffold: intelligent prevention and treatment of infection of bone implants and promotion of bone repair

**DOI:** 10.1016/j.mtbio.2025.102558

**Published:** 2025-11-14

**Authors:** Jiahao Fu, Hai Lan, Likun Liu, Lingling Zou, Xiang Huan, Chenglin Liu, Yaokun Wu, Hao Chen, Hongzhong Xi, Yixuan Huang, Xiaohong Jiang, Guangquan Sun, Xin Liu, Dong Li

**Affiliations:** a Affiliated Hospital of Nanjing University of Chinese Medicine, Nanjing, Jiangsu, 210029, China; b Department of Orthopaedics, Jiangsu Provincial Hospital of Traditional Chinese Medicine, Nanjing, Jiangsu, 210029, China; c ShenShan Hospital, The First Affiliated Hospital of Guangzhou University of Chinese Medicine, Shanwei, 516600, Guangdong, China; d School of Chemistry and Chemical Engineering, Nanjing University of Science and Technology, Nanjing, Jiangsu, 210094, China

**Keywords:** Infection of bone implants, Biomimetic scaffold, Injectable hydrogel, Hyaluronic acid, Infection prevention

## Abstract

Addressing two major challenges in the field of bone implants: bacterial infection and delayed bone fusion, this study draws inspiration from the beehive structure to develop an adaptive enzyme-responsive hydrogel composite scaffold. The scaffold composed of an intermittently 3D low-temperature deposition printing honeycomb-shaped Poly(lactic-co-glycolic acid)/β-tricalcium phosphate icaritin (PTI) outer layer, maintaining the mechanical strength(elastic modulus ∼0.8 MPa) and osteogenic activity required for bone defect repair. Its core encapsulates hyaluronic acid (HA) hydrogel loaded with vancomycin(Van), serving as an intelligent "enzyme-responsive" unit. This core design utilizes bacterial-secreted hyaluronidase (HAase) at the infection site as a trigger signal, enabling on-demand, intelligent release of Van akin to the beehive sensing threats, while degrading slowly in the absence of infection, thereby achieving precise prevention of post-implantation infections. In vitro and in vivo results confirmed the scaffold's excellent dynamic responsive antibacterial performance, effectively eliminating target pathogens. Simultaneously, the hydrogel inner layer exhibited outstanding biocompatibility, synergistically promoting the proliferation, migration, and in vitro osteogenic differentiation of bone marrow mesenchymal stem cells(BMSCs). In a rabbit tibial defect infection model, the scaffold successfully mimicked the beehive's active defense mechanism, effectively suppressing Methicillin-resistant *Staphylococcus aureus* (*MRSA*) infection within 14 days post-implantation, achieveing simultaneous and ideal bone repair outcomes.

## Highlights

• A honeycomb-shaped PTI scaffold was first fabricated using an intermittent 3D low-temperature deposition printing technique.
• VH hydrogel enables hyaluronidase-triggered antibiotic release by bacteria for intelligent on-demand control.
• VH-PTI composite scaffold is fabricated from accessible materials via a simple process and shows favourable biocompatibility.
• Mimicking honeycomb's defense-regeneration, the VH-PTI scaffold enables intelligent infection prevention and bone repair.

## Graphical abstract

(A) Fabrication of the injectable, enzyme-responsive VH hydrogel. (B) Construction of the porous PTI scaffold via an intermittent 3D low-temperature deposition printing strategy. (C) The intelligent responsive mechanism upon implantation in a rabbit tibial defect model: Under physiological conditions (left), the scaffold provides mechanical support and promotes bone regeneration while undergoing slow degradation. In an infected microenvironment (right), HAase secreted by bacteria triggers the rapid, on-demand release of Van from the VH hydrogel core, achieving targeted antibacterial efficacy without compromising the bone repair process.

**Figure:**
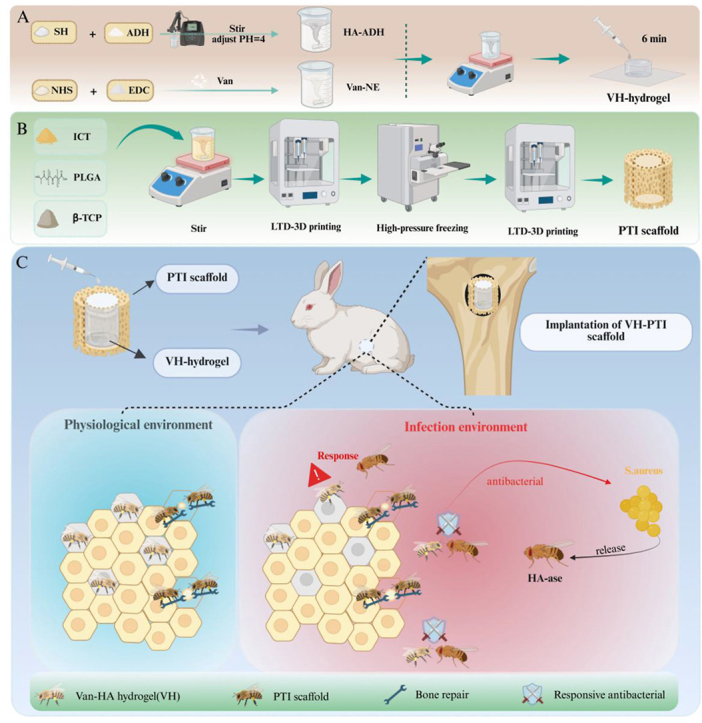

## 1 Introduction

In the field of bone implants, bacterial infection and poor osseointegration remain two major challenges in bone repair [1,2]. These are often interrelated: infection can lead to impaired osseointegration and implant loosening, while delayed osseointegration further increases the risk of infection. Current infection control strategies primarily rely on systemic or local antibiotic therapy. However, the long-term, high-dose application of potent antibiotics like vancomycin (Van) often leads to significantly increased hepatorenal and ototoxicity, and the emergence of drug resistance poses a major challenge [[3], [4], [5]]. To prevent excessive drug release, most antibacterial research has focused on sustained-release systems, such as: biodegradable porous polyurethane (PUR) scaffolds enabling sustained Van release (over 8 weeks), and composite systems integrating Van hydrochloride, recombinant human bone morphogenetic protein-2 (rhBMP-2), and nanoporous magnesium-zinc-silicon (n-MZS) for dual-drug controlled release [[6], [7], [8]]. However, sustained-release systems still suffer from mismatched release kinetics: premature release reduces local effective concentration, while delayed release impedes the osseointegration process. Therefore, there is an urgent need to develop an "intelligent" carrier capable of sensing infection signals in real-time, dynamically regulating drug release, and not disrupting the bone regeneration microenvironment.

Hydrogels are three-dimensional (3D) polymer networks, typically formed through covalent, non-covalent, or physical crosslinking of precursors [9]. In recent years, hydrogels have served as drug delivery platforms, enabling sustained and controlled release of therapeutic agents, thereby targeting drug deposition at injury sites to promote healing [10]. Smart hydrogels based on natural polymers, such as chitosan [11] and hyaluronic acid (HA) [12], and their modified derivatives, have attracted significant attention in biomedical applications due to their excellent antibacterial properties and abundant modifiable functional groups. Notably, the pathogenic bacteria causing bone implants are predominantly Gram-positive [13,14], which produce a metabolic product closely related to diffusion – hyaluronidase (HAase) [15,16]. The activity level of this enzyme is positively correlated with infection severity [[17], [18], [19], [20]]. Leveraging this characteristic, HA hydrogels hold promise as ideal "smart" infection-targeted drug delivery carriers. However, their poor mechanical properties prevent them from meeting the mechanical strength required during osteogenesis, a common limitation of natural polysaccharide-based hydrogels.

In the field of bone repair research, poly(lactic-co-glycolic acid)/β-tricalcium phosphate icaritin (PTI) bioactive scaffolds have garnered widespread attention due to their excellent biocompatibility, degradability, sufficient mechanical properties, and osteogenic activity [[21], [22], [23]]. Nevertheless, lacking antibacterial properties, PTI scaffolds are susceptible to bacterial colonization and cannot effectively prevent or treat implant site infections, posing limitations in practical applications and significantly increasing the risk of post-implantation infection.

In nature, the honeycomb structure provides an excellent biomimetic model for addressing the aforementioned bottlenecks: its outer layer, composed of dense hexagonal wax walls, provides mechanical support and a physical barrier, while the inner layer stores propolis. Under normal conditions, worker bees continuously repair the beehive; when under attack, bees within the beehive can precisely combat invaders while beehive repair remains uninterrupted.

Inspired by this, we propose a biomimetic beehive-like VH-PTI composite scaffold. Its outer layer utilizes an icaritin (ICT)-functionalized PTI material fabricated via intermittent 3D printing (Graphical Abstract b), employing a "3D low-temperature deposition printing-high pressure freezing-3D low-temperature deposition printing" intermittent printing method to endow the scaffold with a hollow honeycomb-like structure. The core consists of an injectable HA hydrogel loaded with Van (Graphical Abstract a). Van is loaded into the 3D network via hydrogen bonding with HA, forming a gel rapidly within 6 min at room temperature. The release rate of Van can be controlled based on HAase concentration, thereby imparting responsive antibacterial properties to the PTI scaffold. Furthermore, its preparation process is straightforward, utilizing readily available materials including sodium hyaluronate (SH), adipic acid dihydrazide (ADH), N-hydroxysuccinimide (NHS), and 1-ethyl-3-(3-dimethylaminopropyl)carbodiimide (EDC). This adaptively enzyme-response composite scaffold, through its biomimetic "beehive wall-core" design, simultaneously fulfills three key requirements: 1. The honeycomb-like PTI scaffold mimics the beehive outer wall, providing osteoconductivity and mechanical support. 2. The Van-loaded HA hydrogel acts as "propolis", utilizing the HAase-triggered mechanism to achieve on-demand drug release, enabling intelligent regulation: "release upon infection, cease upon subsidence". 3. The VH hydrogel mimics the extracellular matrix microenvironment, synergizing with the PTI scaffold to recruit bone marrow mesenchymal stem cells (BMSCs) and promote osteogenic differentiation. Additionally, through in vitro and in vivo evaluations, we systematically analyzed the cytocompatibility, adaptive antibacterial efficacy, and osteogenic promotion capability of the VH-PTI composite scaffold. As shown in Graphical Abstract c, in rabbit tibial defects, this biomimetic structural design successfully mimics the adaptive defense-regeneration mechanism of a beehive, achieving the dual advantages of intelligent infection prevention/treatment and bone regeneration promotion. It can thus be considered a promising drug delivery system for preventing infection of bone implants.

## 2 Materials and methods

### 2.1 Materials

Sodium hyaluronate (SH) [(C_14_H_20_NO_11_Na)_n_, purity ≥95 %, Mw = 200 kDa], Adipic acid dihydrazide (ADH) (purity ≥98 %, DS: 0.25), N-Hydroxysuccinimide (NHS) (purity ≥98 %), 1-Ethyl-3-(3-dimethylaminopropyl) carbodiimide (EDC) (purity ≥98 %), Icaritin (ICT) (HPLC ≥98 %, Mw = 386.4 Da), and Hyaluronidase (HAase) (300 IU/mg) were sourced from Yuanye Biotechnology Co., Ltd. (Shanghai, China). Vancomycin hydrochloride (Van) (purity ≥95 %, Mw = 1485.71 Da) was provided by Meilun Biotech Co., Ltd. (Dalian, China). Poly(lactic-co-glycolic acid) (PLGA) (LA:GA = 50:50; Mw = 48 kDa) and β-Tricalcium phosphate (β-TCP) powders (purity ≥98 %) were obtained from Regenovo Biotechnology Co., Ltd. (Hangzhou, China).

Dexamethasone, Ascorbic acid, β-Glycerophosphate, Dimethyl sulfoxide (DMSO), Cell Counting Kit-8 (CCK-8), Live/Dead viability assay kit, Alkaline phosphatase (ALP) staining kit, RIPA lysis buffer, BCA protein assay kit, Polyvinylidene fluoride (PVDF) membranes, and 4 % paraformaldehyde solution were supplied by Beyotime Biotechnology (Shanghai, China). Dulbecco's Modified Eagle Medium (DMEM) was purchased from Biological Industries (Kibbutz Beit Haemek, Israel). Fetal bovine serum (FBS), Simulated body fluid (SBF), Phosphate-buffered saline (PBS), and 1 % penicillin-streptomycin solution were acquired from Gibco Life Technologies (Grand Island, NY, USA). Primary antibodies against and corresponding secondary antibodies were purchased from Abcam (Cambridge, MA, USA). Antibodies against BMP-2 and β-actin were obtained from Proteintech Group (Wuhan, China). 4′,6-Diamidino-2-phenylindole (DAPI), Cy3-labeled goat anti-rabbit IgG (H + L), FITC-labeled goat anti-rabbit IgG, LB broth (sterile), and immunofluorescence reagents were procured from Beyotime Biotechnology (Jiangsu, China). Hematoxylin and eosin (H&E) staining kits were sourced from Pinuofei Biological Co. (Wuhan, China). Cell culture dishes, multiwell plates, and agar Petri dishes were provided by NEST Biotechnology Co., Ltd. (Wuxi, China). All scaffolds were sterilized using ethylene oxide at 50 °C, following clinical sterilization protocols.

### 2.2 Adipic acid dihydrazide-functionalized hyaluronic acid (HA-ADH)

The aqueous HA-ADH solution was prepared as follows: First, 1.0 g of SH was dissolved in 20 mL of deionized water and stirred continuously at 1000 rpm for 30 min at room temperature. Subsequently, 0.25 g of ADH was slowly added to the mixture. The pH was adjusted to 4 by dropwise addition of 1 M HCl buffer solution under continuous stirring. Finally, vacuum degassing was performed to remove entrapped air bubbles.

### 2.3 Preparation of VH hydrogel

The crosslinking initiator solution was prepared as follows: 0.2 g of NHS and 0.5 g of EDC were dissolved in 1 mL of PBS, vortex-mixed at 3000 rpm, then vacuum degassed and stored at −4 °C. For hydrogel preparation, the Van-containing crosslinker solution was uniformly mixed with the HA-ADH solution under magnetic stirring (1000 rpm, 30 min at room temperature), ultimately yielding an injectable VH hydrogel. The final concentrations were: Van content 0–8 % w/v (specifically 0 %, 0.5 %, 1 %, 2 %, 4 %, 8 %) and HA content 1–7 % w/v (specifically 1 %, 3 %, 5 %, and 7 %).

### 2.4 Characterization of VH hydrogel

#### 2.4.1 Gelation time

The gelation time was determined using the inverted vial test. Briefly, HA-ADH solution and crosslinking initiator solution were thoroughly mixed in a glass vial and immediately incubated at 37 °C. The gelation endpoint was defined as the time required for the mixture to cease flowing upon vial inversion.

#### 2.4.2 Rheological analysis

Viscoelastic properties were evaluated using a rotational rheometer (MCR302). Storage modulus (G′) and loss modulus (G″) were measured under oscillatory shear conditions (frequency: 1 Hz; duration: 570 s) at 37 °C.

#### 2.4.3 Swelling behavior

Swelling behavior was characterized gravimetrically to evaluate drug loading capacity. Lyophilized hydrogels (Ø12 mm × 10 mm height) were immersed in 50 mL of SBF buffer (pH 7.4, 37 °C) to simulate the physiological environment. At predetermined time points, the hydrogels were removed, surface moisture was gently removed using filter paper, and they were weighed until mass equilibrium was reached. Each measurement was performed in triplicate. The swelling ratio (SR) was calculated using the formula: SR (%) = [(W_t_ - W_0_)/W_0_] × 100, where W_0_ represents the initial dry weight and W_t_ is the weight after swelling at time t.

#### 2.4.4 Structural characterization

To analyze hydrogel structure, HA and VH hydrogels were freeze-dried using a lyophilizer (LGJ-10C, Beijing) at −70 °C for 72 h. The lyophilized samples were sputter-coated with gold (20 nm thickness) and then imaged using a scanning electron microscope (SEM, Hitachi Regulus 8100, Japan) to evaluate their internal morphology and drug loading effect. Additionally, two separate sets of VH hydrogels were incubated in SBF solution for 7 and 14 days, respectively, and their microstructural changes were observed using SEM to assess the time-dependent structural evolution.

#### 2.4.5 Enzyme-responsive behavior

Four groups of cylindrical VH hydrogels (Ø20 mm × 10 mm height) were incubated at room temperature in either phosphate-buffered saline (PBS) or HAase solution (concentrations: 0.2, 0.5, 1 mg/mL). Samples were collected at 0, 2, 6, and 10 h for mass determination to quantify degradation kinetics. To further assess enzymatic sensitivity, six additional hydrogel samples were treated with either PBS or 1 mg/mL HAase for 0, 6, and 12 h. After incubation, samples were rinsed, vacuum-dried, and observed via SEM to examine microstructural changes induced by enzymatic digestion.

#### 2.4.6 Dynamic light scattering (DLS) technique

The hydrodynamic diameter and zeta potential of the HA and VH hydrogels were measured using DLS and laser Doppler micro-electrophoresis, respectively. The measurements were performed on a Zetasizer Nano ZS (Malvern Panalytical, UK) at 25 °C. Prior to testing, the hydrogel samples were diluted with deionized water.

### 2.5 Preparation and characterization of VH-PTI composite scaffold

#### 2.5.1 Composite scaffold assembly

The preparation of the hollow honeycomb-like PTI bioactive scaffold followed these specific steps: First, PLGA and β-TCP powder (mass ratio 4:1) were magnetically stirred in 1,4-dioxane at 37 °C for 12 h to form a homogeneous suspension. Subsequently, material containing 2 wt% ICT was added to the mixture. The composite ink was extruded into a mold using a Bio-3D printer (Regenovo Biotechnology Co., Ltd., China) under controlled conditions (nozzle temperature: −28 °C) to preserve ICT bioactivity. The prepared scaffold underwent high-pressure freeze-drying for 24 h, followed by repetition of the 3D printing step. After shaping, the scaffold was stored at −20 °C for later use. For assembly, the VH hydrogel was injected into the hollow channels of the PTI scaffold using a sterile syringe, followed by incubation at 37 °C for 30 min, ultimately forming the honeycomb-like composite structure.

#### 2.5.2 Structural and mechanical characterization

The scaffold morphology was analyzed using scanning electron microscopy (SEM). Uniaxial compression testing was performed using a universal testing machine (Instron 3367, USA) at a crosshead speed of 4 mm/min. After grinding the scaffolds into fine powder, Fourier transform infrared (FTIR) spectroscopy (Thermo Fisher Scientific NICOLET IS20) was employed to collect spectra within the wavenumber range of 500–4000 cm^−1^ to characterize the chemical bonds and features of the synthesized material.

#### 2.5.3 Analysis of intermolecular interactions using the independent gradient model (IGMH)

The Independent Gradient Model based on Hirshfeld partitioning (IGMH) analysis was used to visualize the molecular interactions between the VH hydrogel and the PTI scaffold. The cluster representing the optimal configuration was selected from 1000 configurations generated via rigid conformational searching using Molclus (molclus_1.12_Win) combined with xtb-gfn-1. Wavefunction information was then obtained through energy calculations performed with Gaussian 09 [24]. IGMH analysis, based on Hirshfeld partitioning, was applied to visualize the fragment-fragment interactions [25]. Subsequently, the core-valence bifurcation (CVB) index was used to quantify hydrogen bond strength [26]. Hirshfeld surface analysis and fingerprint plots were also employed to reveal weak interactions. These analyses were performed using Multiwfn 3.8 with visualization aided by VMD 1.9.3 [27].

### 2.6 Van release from the VH-PTI composite scaffold

VH-PTI composite scaffolds were sealed in dialysis bags containing 1 mL of release medium (PBS or HAase solution: 0.2, 0.5, 1 mg/mL). The bags were then immersed in 5 mL of the corresponding solution within a constant-temperature shaker operating at 37 °C and 70 rpm. At predetermined time points, 1 mL samples were withdrawn and replaced with fresh medium. The drug concentration was determined using ultraviolet–visible (UV–Vis) spectrophotometry (Thermo Fisher Scientific, USA) at a wavelength of 280 nm, with background correction using blank solutions. The cumulative drug release was calculated based on a pre-established standard curve.

### 2.7 In vitro biological evaluation

#### 2.7.1 In vitro antibacterial evaluation

Gram-positive *Staphylococcus aureus* ([ATCC] 6538; Shanghai Biolo Engineering Co., Ltd., Shanghai, China) and methicillin-resistant *Staphylococcus aureus* (*MRSA* [ATCC] 43300; Shanghai Biolo Engineering Co., Ltd., Shanghai, China), and *Enterococcus faecalis* (*E. faecalis* [ATCC29212]; Shanghai Biolo Engineering Co., Ltd., Shanghai, China), were inoculated in LB broth and cultured at 37 °C for 24 h. The bacterial suspensions were adjusted to concentrations of 10^4^, 10^5^, 10^6^, 10^7^, 10^8^, and 10^9^ colony-forming units per milliliter (CFU/mL) using sterile broth for subsequent experiments. The minimum inhibitory concentration (MIC) of Van against the *MRSA* strain was determined using the broth microdilution method according to CLSI guidelines. The minimum biofilm eradication concentration (MBEC) was assessed using a crystal violet staining assay on pre-formed biofilms. The detailed procedure was as follows: 200 μL of MRSA suspension (10^6^ CFU/mL) was added to 96-well plates along with Van at varying concentrations, followed by 24-h co-incubation at 37 °C. After incubation, the plates were washed twice with PBS. The samples were then fixed by drying in a 60 °C oven, stained with 0.1 % crystal violet solution for 5 min, washed and dried again, and subsequently decolorized with 33 % glacial acetic acid for 10 min. The OD was measured at 595 nm using a microplate reader, with all samples tested in triplicate.

#### 2.7.2 Evaluation of bacterial HAase release activity

Prior to assessing the effect of the two bacterial species on the hydrogels, the activity of HAase released by bacteria at different concentrations was evaluated by monitoring hydrogel mass changes. Seven groups of VH hydrogels (mass: 1.4 ± 0.2 g) were incubated in HAase solutions with concentrations of 0, 0.05, 0.1, 0.2, 0.5, 1, and 2 mg/mL at room temperature for 12 h. Similarly, six groups of VH hydrogels (mass: 1.4 ± 0.2 g) were incubated in suspensions containing different concentrations of *S. aureus, MRSA* and *E. faecalis* at room temperature for 12 h. The mass changes of the hydrogels over 12 h were recorded to assess the HAase release activity corresponding to the bacterial concentrations.

#### 2.7.3 Plate colony counting

To evaluate the smart drug release efficacy of the composite scaffold, VH-PTI composite scaffolds were incubated in 2 mL of HAase solution (concentrations: 0, 0.2, 0.5, 1 mg/mL) at 37 °C for 2 h. Subsequently, 2 mL of the incubated solution was mixed with 2 mL of *S. aureus* or *MRSA* suspension (concentration: 10^8^ CFU/mL). Control groups were treated with bacterial suspension only. After 24 h of incubation, 100 μL aliquots were spread onto LB agar plates. Following an additional 24 h of culture, colonies were quantified using ImageJ software or the serial dilution method.

The antibacterial efficiency of the VH-PTI composite scaffold was compared with that of polymethylmethacrylate bone cement (PMMA). Van-PMMA (containing 4 % w/v Van, 200 μL solid cement) or the VH-PTI composite scaffold (200 μL hydrogel) was incubated with 2 mL of SBF solution at 37 °C. The SBF solution was replaced daily, and the incubation media from days 1 and 7 were retained. On days 11 and 13, samples were treated with HAase (0.2 mg/mL) for 2 h each day. On day 14, the treatment was switched to 1 mg/mL HAase for 2 h, while the control group received no treatment. Subsequently, the incubation media from each group were mixed with 2 mL of *MRSA* suspension (concentration 10^8^ CFU/mL) and co-incubated for 24 h. Then, 100 μL samples were spread evenly on LB agar plates, followed by another 24 h of incubation. Colonies were quantified using ImageJ software or the serial dilution method.

#### 2.7.4 Live/dead bacterial staining

Bacterial viability was assessed using the Live/Dead staining kit (Life Technologies Corporation, CA). Briefly, 2 mL of bacterial suspensions at different concentrations were transferred into 24-well plates and co-cultured with various scaffold materials at 37 °C for 24 h. Untreated suspensions served as the control. After incubation, the original bacterial suspension was discarded, replaced with the working staining solution according to the manufacturer's instructions, and incubated. Subsequently, 10 μL samples were aspirated and observed under an inverted fluorescence microscope to visualize the distribution of live bacteria (green fluorescence) and dead bacteria (red fluorescence).

#### 2.7.5 Inhibition zone assay

To evaluate the enzyme-responsive antibacterial capability of the VH-PTI composite scaffold against bacteria at different concentrations, an inhibition zone assay was performed. Specifically, 100 μL of *S. aureus* or *MRSA* suspensions at concentrations of 10^4^, 10^6^, and 10^8^ CFU/mL were spread evenly onto agar plates, while *E. faecalis* was cultured on blood agar medium. Sterilized scaffolds were then placed onto the plates, followed by incubation at 37 °C for 24 h. HA-PTI scaffolds without Van served as the control group. After incubation, images were captured, and the diameter of the inhibition zone was measured. The experiment was performed in triplicate. To further investigate the dynamic responsive antibacterial activity of the VH hydrogel, the agar well diffusion method was employed to assess inhibition zone sizes against different bacterial concentrations. Following the bacterial spreading procedure described above, uniform wells were created in the center of agar plates using a 10 mL pipette tip. Subsequently, 200 μL of VH hydrogel was added to each well, and plates were incubated at 37 °C for 24 h. Finally, images were captured to observe the inhibition zone size and hydrogel degradation state.

### 2.8 In vitro cell experiments

Rabbit bone marrow mesenchymal stem cells (BMSCs) used in the experiments were purchased from the Shanghai Cell Bank, Chinese Academy of Sciences (Shanghai, China). Cells were cultured in complete medium consisting of 89 % DMEM, 10 % fetal bovine serum (FBS), and 1 % penicillin-streptomycin, maintained at 37 °C in a humidified atmosphere of 5 % CO_2_ (Henghe Biological Incubator, China). The medium was changed every 48 h. Cell growth was monitored using an Olympus microscope. Cells were passaged when reaching 80–90 % confluence, and passage 3 (P3) cells were selected for subsequent experiments.

#### 2.8.1 Cytocompatibility of VH hydrogel

To assess the dose-dependent effect, BMSCs (5 × 10^3^ cells per well) were seeded in the lower chambers of 96-well Transwell plates. Then, 50 μL of VH hydrogel containing different Van concentrations (0, 0.5, 1, 2, 4, 8 % w/v) was placed in the upper Transwell inserts. After 24 and 48 h of incubation, cell proliferation was evaluated using the CCK-8 kit according to the manufacturer's instructions. Absorbance (OD) at 450 nm was measured using an automated enzyme marker instrument (BioTek Instruments, USA) to assess cell viability. Each experiment was performed in triplicate.

#### 2.8.2 Biocompatibility of VH-PTI composite scaffold

BMSCs (5 × 10^3^ cells/well) were co-cultured with PTI or VH-PTI scaffolds in 96-well Transwell plates using complete medium. Wells without scaffolds served as the blank control. After 24 and 48 h of incubation, cell viability was detected using the CCK-8 assay. Each group was tested in triplicate. Parallel samples were stained using the Live/Dead Cell Viability Assay Kit (Calcein AM/PI): cells were rinsed with PBS and then incubated with the dye solution in the dark at 37 °C for 15 min. Dead cells exhibited red fluorescence, while live cells displayed green fluorescence. Images of stained cells were captured using an inverted fluorescence microscope (Olympus, Japan).

#### 2.8.3 EdU staining

To analyze cell proliferation, after seeding and grouping cells as described above and culturing for 24/48 h, EdU staining was performed using the BeyoClick™ EdU Cell Proliferation Kit (Beyotime, Shanghai, China). Cells were first washed with PBS. Fresh DMEM containing 10 μM EdU was then added to the medium. Following a 2-h incubation at 37 °C/5 % CO_2_, cells were washed again with PBS to remove DMEM and free EdU probes. Cells were then fixed in 4 % paraformaldehyde at room temperature for 30 min, followed by staining with 4′,6-diamidino-2-phenylindole (DAPI) for 3 min. After additional washing in PBS, cells were observed under an inverted fluorescence microscope.

#### 2.8.4 Cell migration assay

The migratory capacity of bone marrow mesenchymal stem cells (BMSCs) was assessed using a transwell assay. BMSCs suspended in serum-free medium (1 × 10^4^ cells/insert) were seeded into the upper chambers of 24-well transwell plates. The lower chambers contained complete medium with either PTI/VH-PTI scaffolds or control groups. After 24 h, non-migrated cells on the upper surface of the membrane were removed using cotton swabs. Migrated cells on the lower surface were fixed (4 % paraformaldehyde, 30 min), stained with 0.1 % crystal violet (Solarbio, China) for 20 min, and imaged using an inverted phase-contrast microscope (Olympus, Japan). Migrated cells in three random fields per chamber were counted using ImageJ software.

The effect of different scaffold groups on the horizontal migration capacity of BMSCs was investigated using a scratch wound assay: BMSCs (2 × 10^5^ cells/well) were cultured in 6-well transwell plates until adherent. A uniform scratch wound was created using a 200 μL pipette tip. Scaffolds were then placed in the upper chamber, while the lower chamber received medium containing 1 % FBS. Wound closure was monitored at 0 and 24 h using the inverted phase-contrast microscope, and the remaining scratch area was measured using ImageJ.

#### 2.8.5 Alkaline phosphatase (ALP) and Alizarin Red S (ARS) staining

BMSCs (2 × 10^4^ cells/well) were seeded in the lower chambers of 24-transwell transwell plates. Upon reaching 70–80 % confluence, the medium was replaced with osteogenic induction medium (OIM: DMEM supplemented with 10 % FBS, 1 % penicillin-streptomycin, 50 μM ascorbic acid, 100 nM dexamethasone, and 10 mM β-glycerophosphate). After 7/14 days of co-culture with scaffolds in the upper chambers, ALP and ARS staining were performed using the BCIP/NBT kit (Beyotime, China) and ARS (pH 4.2, Solarbio, China), respectively. Stained samples were gently rinsed with ddH_2_O buffer, observed under an inverted phase-contrast microscope, and quantitatively analyzed using ImageJ software (three random regions of interest per sample).

#### 2.8.6 Immunofluorescence staining for osteogenic-related proteins

BMSCs (2 × 10^5^ cells/well) were co-cultured with scaffold materials in 6-well transwell plates for 120 h. Cells were fixed using an immunofluorescence fixative for 10 min at room temperature. After two washes with immunofluorescence washing buffer, cells were blocked with immunofluorescence blocking buffer for 1 h, followed by incubation with primary antibodies (BMP-2, ALP, Runx2; dilution 1:500) overnight at 4 °C. After removing excess primary antibody, corresponding secondary antibodies were added and incubated for 1 h. Subsequently, BMSC nuclei were stained and visualized using DAPI. Images of stained cells were captured using an inverted fluorescence microscope (Olympus, Japan) to observe the location and intensity of target proteins, and signal intensity was quantified using ImageJ software.

#### 2.8.7 RNA extraction and real-time quantitative PCR (RT-qPCR)

Total RNA was extracted from cells and reverse-transcribed into cDNA. Gene-specific primers (Table S4) were synthesized by Wuhan Saier Biotechnology. qPCR was performed using SYBR Green chemistry. The relative mRNA expression levels of target genes were normalized to a housekeeping gene and calculated using the 2-ΔΔCt method. All experiments were performed with six independent replicates (n = 6).

#### 2.8.8 Western blot analysis

Total protein was extracted from BMSCs co-cultured with scaffolds (cultured for 120 h) using RIPA lysis buffer. Protein extracts were diluted with protein loading buffer (5 × , NCM Biotech, China), and equal amounts of protein from each group were subjected to sodium dodecyl sulfate-polyacrylamide gel electrophoresis (SDS-PAGE). Target proteins were then transferred onto polyvinylidene fluoride (PVDF) membranes. The PVDF membranes were incubated with corresponding primary antibodies against (Table S5) overnight at 4 °C, followed by incubation with secondary antibodies (1:500) at room temperature for 1 h. Target protein bands were detected and quantified using a chemiluminescence imaging analyzer (Bio-Rad, USA). Band intensity was normalized to β-actin using ImageJ software.

### 2.9 In vivo rabbit experiments

#### 2.9.1 Animal model establishment

Animal experiments strictly followed the guidelines for animal use and were approved by the Animal Ethics Committee of the Affiliated Hospital of Nanjing University of Chinese Medicine (Approval No.: 2025NL-KS021). Three-month-old female New Zealand white rabbits weighing 2.6 ± 0.15 kg were used. Rabbits were housed individually under controlled environmental conditions (20 °C ± 1 °C, 12-h light/12-h dark cycle). A tibial tuberosity infection model was established as previously reported [28]. Forty rabbits were randomly assigned to a control group (n = 10) and an experimental group (n = 30), with the latter further subdivided into three subgroups (n = 10 each) based on *MRSA* inoculum concentration. After drilling a 6 mm diameter defect in the left tibial tuberosity, all rabbits received different treatments: The control group received an implant of *MRSA* (200 μL, 1 × 10^6^ (CFU)/mL) into the defect site, followed by insertion of a PTI scaffold. The experimental subgroups received an implant of a precisely quantified 200 μL MRSA suspension at concentrations of 0 CFU/mL, 1 × 10^6^ CFU/mL, or 1 × 10^8^ CFU/mL, respectively. The bacterial inoculum was prepared from mid-logarithmic phase cultures, and its concentration was verified by plate counting prior to surgery. The suspension was injected directly into the defect site immediately before scaffold implantation, followed by insertion of the VH-PTI composite scaffold. The surgical procedure is illustrated in Fig. 8a, and no additional antibacterial treatment was administered.

#### 2.9.2 Evaluation of antibacterial activity

To assess the antibacterial activity of the VH-PTI composite scaffold, inflammatory markers in rabbit serum, including high-sensitivity C-reactive protein (CRP) and white blood cell (WBC) count, were measured at postoperative days 0, 7, and 14. Temperature changes in the tibial tuberosity region were recorded using infrared thermography at days 7 and 14, and body weight changes were monitored weekly for 8 weeks. At the end of weeks 1 and 2, rabbits were euthanized via an overdose of sodium pentobarbital. The defect site was then excised, ground, and homogenized in PBS. A 100 μL aliquot of the homogenate was spread onto agar plates. After overnight incubation at 37 °C, the number of CFUs was counted.

#### 2.9.3 Determination of drug concentration in bone tissue

On postoperative days 7, 14, and 28, bone tissue samples surrounding the scaffold were harvested from the experimental groups. A 0.2 g tissue sample was placed in 1.5 mL saline and homogenized using a cryogenic grinder. The homogenate was centrifuged at 6000 rpm for 10 min at 4 °C. The supernatant was then analyzed using UV–Vis spectrophotometry at 280 nm. Van concentration was quantified based on a pre-established standard curve (Fig. S1e).

#### 2.9.4 Micro-CT 3D reconstruction and hematoxylin & eosin (H&E) staining for anti-infection and osteogenic evaluation

Rabbits were euthanized at weeks 4 and 8 post-surgery, and tibial specimens were harvested for in-depth analysis. Specimens were scanned using a high-resolution micro-CT system (Siemens, Germany). Bone repair efficacy was evaluated using Micro-View 3D reconstruction analysis software (GE Healthcare). Subsequently, Hematoxylin and Eosin (H&E) staining was performed. All samples were decalcified in 10 % EDTA solution for 6 weeks, followed by dehydration, clearing, and embedding in paraffin. Sections (5 μm thick) were prepared and imaged using a Nikon optical microscope.

#### 2.9.5 Biosafety evaluation

The hemolytic activity of the materials was determined using a previously reported method [29]. Briefly, a 2 % (v/v) suspension of rabbit red blood cells (RBCs) was co-incubated with VH hydrogel, PTI scaffold, or VH-PTI composite scaffold. PBS served as the negative control and 1 % Triton X-100 as the positive control. Incubation was performed under simulated physiological conditions (37 °C for 4 h). After incubation, the supernatant was centrifuged, and the hemolysis rate was measured at 540 nm using a UV/Vis spectrophotometer. The hemolysis rate (%) was calculated as: Hemolysis Rate (%) = [(As - Ab)/(At - Ab)] × 100, where As is the absorbance of the sample group, Ab is the absorbance of the negative control (PBS), and At is the absorbance of the positive control (Triton X-100). Peripheral blood samples were collected from the marginal ear vein of rabbits in each group before modeling and at postoperative days 7 and 14 for liver and kidney function tests. At weeks 4 and 8 post-surgery, major organs (heart, liver, spleen, lungs and kidneys) were harvested for pathological analysis using H&E staining.

### 2.10 Statistical analysis

Data are expressed as mean ± standard deviation (SD). Multiple group comparisons were performed using one-way analysis of variance (ANOVA), and differences between two groups were determined using *t*-test. Statistical analysis was conducted using GraphPad Prism software (Version 9.0, USA). Statistical significance was defined as *P < 0.05, **P < 0.01, and ***P < 0.001; P > 0.05 was considered not statistically significant.

## 3 Results and discussion

### 3.1 Characterization of VH hydrogel

**Fig. 1:**
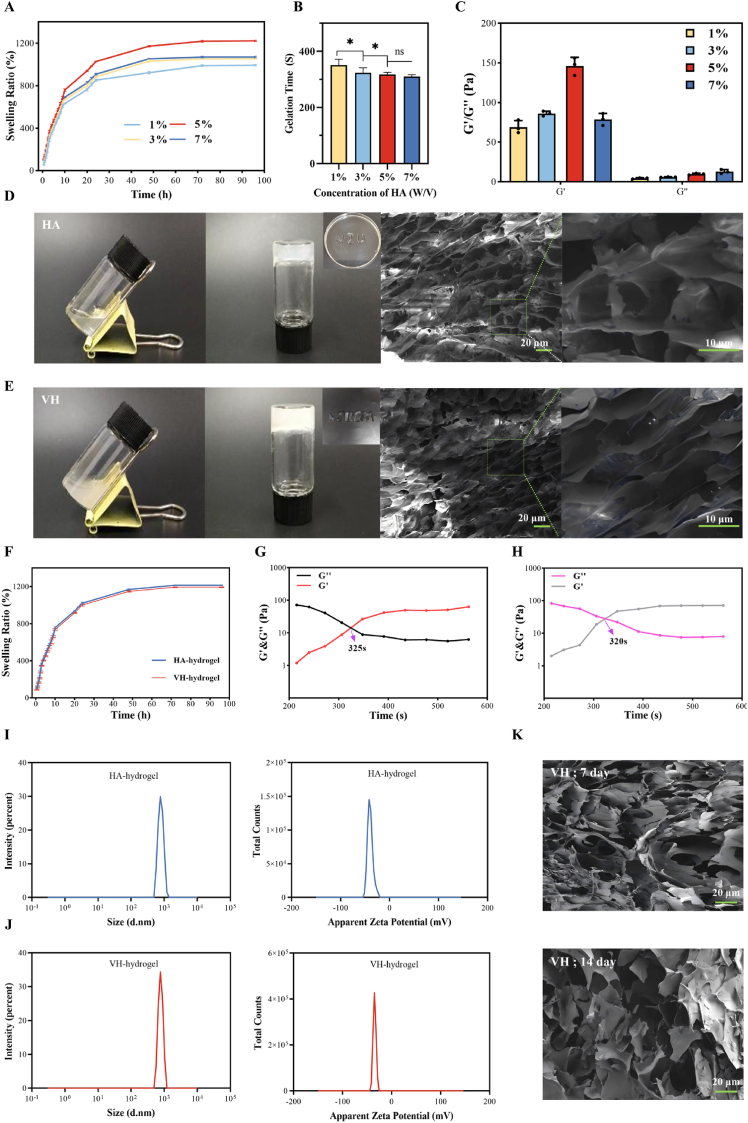
Characterization of HA hydrogels and VH hydrogels. Different concentrations of HA hydrogels (A) swelling ratios, (B) gelation time, and (C) rheological analysis. (D) Solution-gel transition, injectability, and SEM images of HA hydrogels. (E) Solution-gel transition, injectability, and SEM images of VH hydrogels. (F) Swelling ratios of HA hydrogels and VH hydrogels. (G) Rheological properties of HA hydrogels over time. (H) Rheological properties of VH hydrogels over time. (I) Hydrodynamic size and (J) Zeta potential of the HA hydrogel and VH hydrogel precursor solutions. (K) SEM images illustrating the microstructure of the VH hydrogel after immersion in SBF for 7 and 14 days. Data are presented as mean ± SD (n = 3, *p < 0.05).

To optimize the SH concentration in the hydrogel formulation, we systematically evaluated hydrogels with different HA concentrations (1 %, 3 %, 5 %, 7 % w/v) through swelling ratio analysis, rheological property testing, and frequency sweep tests. As shown in Fig. 1a, the 5 % w/v HA hydrogel exhibited excellent swelling capacity (equilibrium swelling ratio reaching 1230 %), indicating superior drug loading potential. Frequency sweep analysis (Fig. 1c) revealed significantly enhanced viscoelastic properties at the 5 % concentration, with the storage modulus (G' = 156 Pa) substantially higher than the loss modulus (G'' = 10 Pa). Rheological analysis (Fig. 1b) further demonstrated that the sol-gel transition kinetics of the 5 % HA hydrogel were significantly accelerated, achieving gelation within 325 s. Based on the comprehensive experimental results, 5 % w/v was determined to be the optimal HA concentration. Fig. 1d–e illustrates the sol-gel transition process, injectability, and scanning electron microscopy (SEM) images of the HA and VH hydrogels. At room temperature, the HA hydrogel completed the sol-gel transition within 325 s, forming an opaque, injectable hydrogel. In contrast, the darker-colored VH hydrogel transitioned slightly faster, requiring only 320 s. SEM analysis showed that the HA hydrogel formed a dense porous network structure, while the VH hydrogel exhibited more densely packed pores. Swelling ratio tests (Fig. 1f) indicated that both hydrogels reached equilibrium swelling after 72 h, with the HA hydrogel achieving 1217 % and the VH hydrogel 1196 %. As shown in Fig. 1g–h, the G′ and G″ values of the VH hydrogel were significantly higher than those of the HA hydrogel. Furthermore, to thoroughly characterize the physicochemical properties, we performed DLS and Zeta potential analyses on the precursor solutions. The results in Fig. 1i–j shows that the VH hydrogel precursor had a larger average hydrodynamic diameter (1419 nm) compared to the HA precursor (1134 nm), suggesting that the incorporation of Van may promote the formation of larger complexes. Both HA and VH precursor solutions exhibited significantly negative Zeta potentials (−39.5 mV and −34.78 mV, respectively), a characteristic feature of HA attributable to its carboxyl groups. This highly negative surface charge enhances the colloidal stability of the system and may improve biocompatibility by reducing non-specific protein adsorption. The slight decrease in the absolute Zeta potential value for the VH hydrogel suggests an interaction between the positively charged groups of Van and the negatively charged HA backbone, providing a structural basis for the formation of a more densely cross-linked network. Collectively, these results demonstrate that the incorporation of Van not only enhanced mechanical strength by increasing crosslinking density but also slightly reduced gelation time, without compromising injectability.

**Fig. 2:**
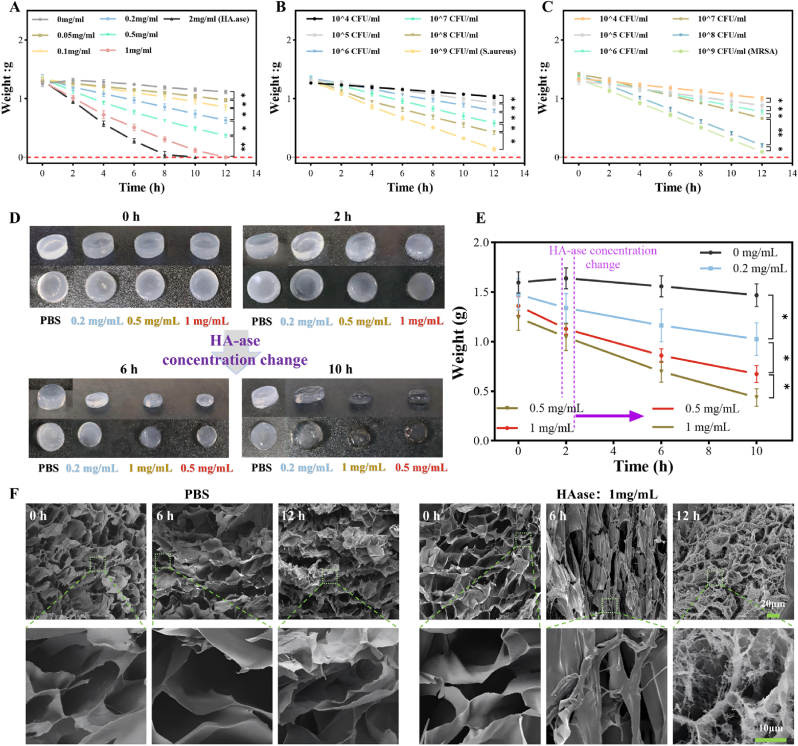
HAase-responsive behavior of VH hydrogel. (A–F) VH hydrogel: Mass variation with different concentrations of (A) HAase (B)S.aureus and (C) *MRSA* co-cultured for 12h; (D) Degradation behavior at different HAase concentrations and (E) mass variation; (F) SEM images after immersion in PBS and 1 mg/mL HAase solution for 0, 6, and 12 h. Data are presented as mean ± SD (n = 3, *p < 0.05, **p < 0.01).

To precisely evaluate the HAase-triggered degradation kinetics, VH hydrogels with an initial mass of 1.4 ± 0.2 g were co-incubated with either HAase solutions at varying concentrations (0, 0.05, 0.1, 0.2, 0.5, 1, and 2 mg/mL) or suspensions of *S. aureus* and *MRSA* at different bacterial concentrations. Degradation was monitored over 12 h. As shown in Fig. 2a–c, the hydrogel degradation rate exhibited a positive correlation with both HAase concentration and bacterial concentration. A similar trend was observed for *E. faecalis* (Fig. S2a), indicating that the VH hydrogel modulates its degradation rate in response to variations in HAase produced by these three bacterial species. To visualize the effect of HAase on the VH hydrogel more directly, VH hydrogels (1.4 ± 0.2 g) were incubated with HAase solutions at concentrations of 0.2, 0.5, and 1 mg/mL and PBS for 10 h, Groups 1, 2, 3 and 4 were named respectively. Results depicted in Fig. 2d–e shows a concentration-dependent mass loss during the initial phase (0–2 h). In contrast, the PBS group1 exhibited gradual weight gain, attributed to incomplete swelling equilibrium. To further investigate the dynamic responsive nature, the HAase concentration was adjusted after 2 h for Groups 3 and 4, while Groups 1 and 2 maintained their initial concentrations. In the subsequent phase (2–10 h), Groups 1 and 2 exhibited persistently slow degradation rates, whereas Groups 3 and 4 showed a distinct reversal in degradation rates. These results confirm the VH hydrogel's ability to dynamically regulate its degradation rate according to HAase concentration, highlighting its smart responsive nature as a drug carrier. SEM images (Fig. 2f) further revealed the progressive erosion of the porous network structure upon treatment with 1 mg/mL HAase for 0, 6, and 12 h. This manifested as surface roughening with micro-void formation and eventual collapse. Conversely, only partial degradation occurred after 12 h of PBS treatment,and the hydrogel's porous network structure remained largely intact with only a slight increase in pore size after 7 days. By day 14, the structure maintained connectivity but exhibited partial pore wall fusion (Fig. 1k), indicating a slow, time-dependent degradation process consistent with the observed swelling and in vitro degradation behavior. Collectively, these findings demonstrate that the VH hydrogel achieves infection-adaptive antibiotic release through HAase concentration-dependent matrix erosion mediated by bacterial secretion, validating its potential as an intelligent drug delivery vehicle.

### 3.2 Preparation and characterization of the VH-PTI composite scaffold

**Fig. 3:**
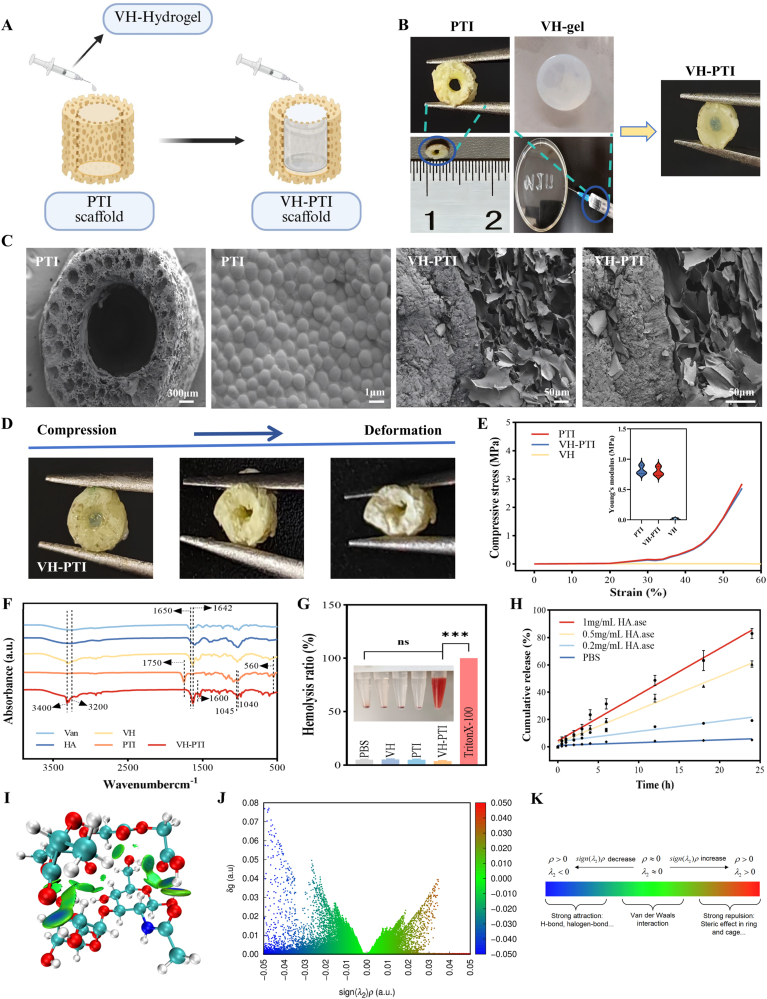
Fabrication and characterization of composite scaffolds. (A) Schematic diagram illustrating the assembly process of the VH-PTI composite scaffold. The PTI scaffold with hollow honeycomb channels is fabricated via intermittent 3D printing. Subsequently, the injectable VH hydrogel precursor solution is infused into the channels using a syringe, where it undergoes rapid in situ gelation at room temperature to form the final composite structure. (B) Appearance and composition of the scaffolds. (C) SEM images of PTI and VH-PTI scaffolds. (D) Image of compression deformation of VH-PTI composite scaffold. (E) Stress-strain curves and Young's moduli of different scaffolds. (F) FTIR spectra of Van, HA hydrogel, VH hydrogel, PTI scaffold, and VH-PTI composite scaffold. (G) Hemolytic test. (H) Van cumulative release in different solutions. (I) the colored function δginter map of HA-PLGA. (J) Sign(λ_2_)ρ colored IGMH scatter plot of HA-PLGA. (K) the color scale bar indicating different interaction. Data are presented as mean ± SD (n = 3,***p < 0.001).

Fig. 3a–b illustrates the experimental schematic and physical overview of the VH-PTI composite scaffold assembly. The honeycomb-like PTI scaffold, fabricated via intermittent 3D printing, measured 3 mm in diameter and 4 mm in height and exhibited a rough surface. To determine the optimal outer-to-inner diameter ratio, scaffolds with ratios of 1:1, 1:2, and 1:4 were subjected to compression testing. Results (Fig. S1a) showed that while the 1:4 ratio scaffold possessed good mechanical strength, its deformability was insufficient. The 1:1 ratio scaffold offered superior deformability but compromised mechanical strength. Conversely, the 1:2 ratio scaffold combined high mechanical strength with favorable deformability. Consequently, the 1:2 ratio scaffold was selected for further experiments (Fig. 3b). The core VH hydrogel (injectable hydrogel, Fig. 3b) was uniformly injected into the internal channels of the PTI scaffold using a syringe, successfully forming the VH-PTI composite scaffold. SEM analysis (Fig. 3c) revealed the honeycomb structure of the PTI scaffold, characterized by a rough surface uniformly covered with regularly arranged PLGA particles, resembling a dense honeycomb. Images of the VH-PTI composite scaffold clearly showed the interconnected interface between the PTI scaffold and the VH hydrogel, similar to the connection between honeycomb and propolis, confirming the successful preparation of the biomimetic honeycomb-structured VH-PTI composite scaffold.

Mechanical properties are crucial in bone defect repair as they provide a stable environment for new bone formation, prevent displacement, and ensure a smooth bone regeneration process [30,31]. As shown in Fig. 3d, the scaffolds undergo significant deformation under compression. To evaluate the mechanical strength of the composite scaffold, mechanical testing was performed on the VH-PTI composite scaffold and compared to the VH hydrogel and PTI scaffold alone. The stress-strain curves (Fig. 3e) indicated that the VH hydrogel possessed low mechanical strength and was prone to collapse under load, exhibiting a linear stress-strain profile. When strain exceeded 30 %, the stress-strain curves of both the PTI and VH-PTI scaffolds plateaued, signifying plastic deformation that persisted until 50 % strain, after which the scaffolds gradually densified. The elastic modulus, calculated from the linear region of the stress-strain curves, was approximately 0.8 MPa for both the PTI and VH-PTI scaffolds, demonstrating excellent performance. These results indicate that both the VH-PTI composite scaffold and the PTI scaffold possess favorable mechanical strength and plasticity, and that hydrogel loading does not compromise scaffold properties. Notably, the elastic modulus falls within the reported "osteogenic induction" range (25–40 kPa), confirming its effectiveness in inducing stem cell osteogenic differentiation [32]. Importantly, numerous studies demonstrate that mesenchymal stem cells exhibit enhanced growth and osteogenic differentiation on substrates with stiffness exceeding this range. For instance, Hu et al. fabricated decellularized bone matrix (DBM) scaffolds with varying stiffness levels. Compared to stiffer DBM, scaffolds with a modulus of 0.67 ± 0.14 MPa not only promoted osteogenesis but also significantly enhanced osseointegration [33]. Chen et al. combined porous bioglass nanoparticles (BGNS) with poly(citrate-siloxane) (PCS) via 3D printing to create BGNS@PCS scaffolds with an elastic modulus of 0.78 MPa and excellent compressive strength. These scaffolds significantly promoted cell proliferation and BMSC osteogenic differentiation [34]. Therefore, compared to high-stiffness matrices, lower-stiffness biomaterials may enhance cellular perception of physical stimuli and promote the transduction of mechanical signals to membrane-bound target proteins, thereby better supporting cell proliferation and enhancing osteoblast differentiation [35]. Based on this evidence, the VH-PTI composite scaffold with an elastic modulus of 0.8 MPa may effectively promote osteoblast differentiation through combined mechanical stimulation and osteogenic signaling.

FTIR spectroscopy was employed to identify the chemical groups in the VH hydrogel, PTI scaffold, and VH-PTI composite scaffold. As shown in Fig. 3f, the coexistence of characteristic peaks from HA and Van in the VH hydrogel was confirmed. Broadening of the hydroxyl peak (shifted from 3200 cm^−1^ to 3400 cm^−1^) and a red shift of the amide I band (shifted from 1650 cm^−1^ to 1642 cm^−1^) indicate hydrogen bonding between the two components. In the PTI scaffold, characteristic peaks corresponding to PLGA (C=O stretching vibration at 1750 cm^−1^), PO_4_^3−^ (bending vibration at 560 cm^−1^), β-TCP (asymmetric PO_4_^3−^ stretching vibration at 1040 cm^−1^), and ICT (C=C skeletal vibration at 1600 cm^−1^) were clearly visible, confirming the scaffold's composition. In the VH-PTI composite scaffold, the characteristic peaks of both VH hydrogel and PTI were fully retained. However, broadening of the hydroxyl peak and a slight shift of the PO_4_^3−^ peak (1040 cm^−1^ → 1045 cm^−1^) suggest non-covalent coordinative interactions between the VH hydrogel and PTI surfaces, ensuring the structural integrity of the components. We further investigated the intermolecular interactions underpinning the coordinative relationship between the VH hydrogel and the PTI scaffold surface. The microscopic binding process between HA and PLGA was simulated. Independent Gradient Model based on Hirshfeld partitioning (IGMH) analysis was used to study the types and strengths of weak interactions. From an atomic perspective, Fig. 3i and j displays the 3D isosurface plot and scatter plot for this system. As shown in Fig. 3k, blue, green, and red regions represent hydrogen bonding, van der Waals (vdW), and steric hindrance interactions, respectively. A distinct sharp peak was observed in δginter at sign(λ_2_)ρ ≈ −0.05 a.u. (δginter ≈ 0.08 a.u.), signifying hydrogen bonding interactions; a peak corresponding to van der Waals (vdW) interactions was observed at sign(λ_2_)ρ ≈ −0.03 a.u. (δginter ≈ 0.05 a.u.); a small peak reflecting steric hindrance was observed at sign(λ_2_)ρ ≈ +0.035 a.u. These weak interactions promote interfacial binding. However, the area of the blue region in the isosurface plot and the intensity of the blue peak in the scatter plot both indicate that hydrogen bonding interactions are dominant. Structurally, this is likely due to hydrogen bonds forming between the hydrogen atoms of hydroxyl (-OH) or carboxylic acid (-COOH) groups in hyaluronic acid and the oxygen atoms of ester groups (-COO-) in PLGA. The introduction of hydrogen bonds and vdW forces can enhance the interfacial binding strength [36,37]. Therefore, hydrogen bonding and vdW forces facilitate the binding between the VH hydrogel and the PTI scaffold, with hydrogen bonding being the most significant factor in this process.

The ability to regulate Van release in response to HAase levels—a virulence factor overexpressed during bacterial infection [16,38,39]—constitutes a key innovation of this study. To evaluate this dynamic responsive property, VH-PTI composite scaffolds were co-incubated with PBS or HAase solutions at different concentrations (0.2, 0.5, 1 mg/mL) at 37 °C. Cumulative drug release was quantified using a pre-established standard curve (Fig. S1e). As shown in Fig. 3h, Van release exhibited a strict HAase concentration-dependence: the fastest release rate and highest cumulative release (83 % at 24 h) occurred at the highest HAase concentration (1 mg/mL). At lower concentrations (0.2 and 0.5 mg/mL) and in PBS, the 24-h cumulative release rates were significantly lower at 60.3 %, 19.8 %, and 5.6 %, respectively. This demonstrates that the VH-PTI composite scaffold enables intelligent, on-demand drug delivery by modulating the release rate according to the HAase concentration.

### 3.3 In vitro antibacterial activity of the VH-PTI composite scaffold

Prior to evaluating the antibacterial efficacy of the VH-PTI composite scaffold, the optimal VH hydrogel concentration was screened using cellular and antibacterial assays. The survival rate of BMSCs in hydrogels containing Van concentrations ranging from 0 to 8 % w/v was assessed. CCK-8 results (Fig. S1b) showed excellent cytocompatibility across all concentration groups (viability >105 % at 24/48 h), primarily attributed to the HA mimicking the porous structure of the extracellular matrix and its bioactive degradation products [40,41]. However, compared to the 4 % w/v group, a significant decrease in cell viability (P < 0.05) was observed at 48 h for the 8 % w/v hydrogel, potentially related to the dose-dependent toxicity of Van towards osteoblasts [42]. Similarly, results from bacterial suspension and inhibition zone assays (Fig. S1c–d) indicated that VH hydrogels with 4 % and 8 % w/v Van exhibited comparable antibacterial efficacy against *S. aureus* at different concentrations (10^4^, 10^6^, 10^8^ CFU/mL), both significantly superior to other concentrations (P < 0.05). Considering the cytocompatibility results, 4 % w/v Van was ultimately selected for subsequent experiments.

**Fig. 4:**
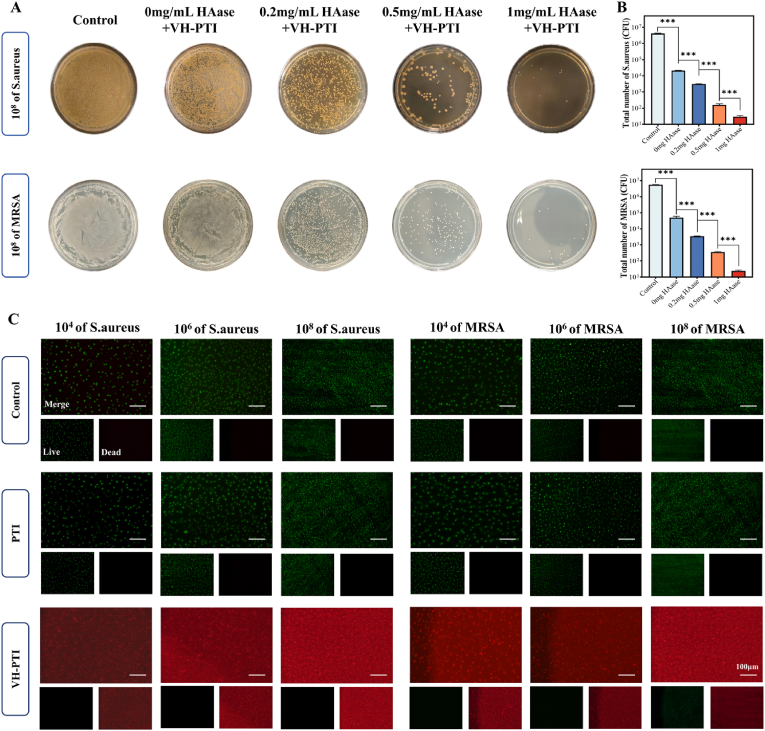
In vitro antibacterial activity. (A) Colony-forming unit assays of *S. aureus and MRSA* after co-culture with scaffolds pretreated with HAase (0, 0.2, 0.5, and 1 mg/mL) for 2 h, and (B) quantitative analysis. (C) Live/dead staining of *S. aureus and MRSA* co-cultured with scaffolds at different concentrations (green: live bacteria, red: dead bacteria). Data are presented as mean ± SD (n = 3,***p < 0.001).

**Fig. 5:**
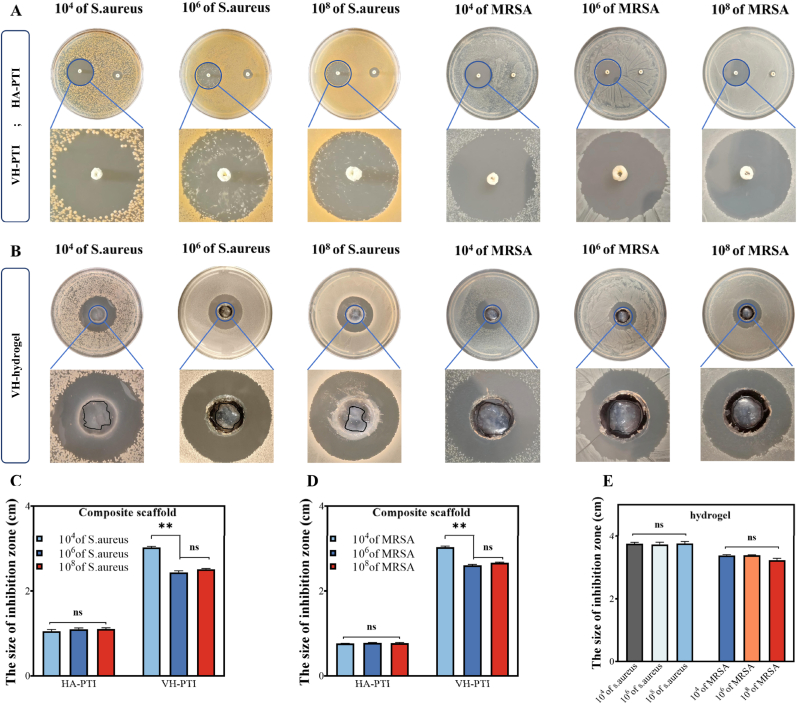
In vitro antibacterial activity. (A) Zone of inhibition assays of HA-PTI and VH-PTI composite scaffolds against *S. aureus and MRSA* at various concentrations, and (C–D) quantitative analysis. (B) Zone of inhibition assays of VH hydrogels against different concentrations of *S. aureus and MRSA* and (E) quantitative analysis. Data are presented as mean ± SD (n = 3,**p < 0.01).

To evaluate the enzyme-responsive antibacterial capability of the VH-PTI composite scaffold, scaffolds were pre-treated with HAase solutions at different concentrations for 2 h before co-culturing with *S. aureus* and *MRSA* suspensions. Results (Fig. 4a–b) demonstrated that bacterial colony counts significantly decreased with increasing HAase concentration compared to the blank control group. At the highest HAase concentration (1 mg/mL), only a few colonies were visible (P < 0.001). The antibacterial effect of the VH-PTI composite scaffold was further assessed via Live/Dead bacterial staining (Fig. 4c). Results showed predominantly live bacteria (green) in the blank and PTI groups, whereas the VH-PTI group exhibited predominantly dead bacteria (red) with minimal live bacteria. This trend remained consistent across different concentrations of *S. aureus* and *MRSA*. The inhibition zone assay (Fig. 5a) showed that the HA-PTI composite scaffold possessed a certain level of antibacterial activity against bacteria at various concentrations. The inhibitory effect of the VH-PTI composite scaffold on bacterial growth was more pronounced, consistent with the Live/Dead staining results. Quantitative analysis of inhibition zone diameters (Fig. 5c–d) revealed no significant difference for the HA-PTI group across bacterial concentrations, with an average diameter of approximately 8 mm. This might be attributed to the combined effect of HA and HAase produced by bacteria limiting bacterial invasion. For the VH-PTI group, no significant difference in inhibition zone diameter was observed between 10^6^ and 10^8^ CFU/mL, but a significant difference existed compared to 10^4^ CFU/mL (P < 0.01). Fig. 5b and e visually illustrate the dynamic responsiveness of the intelligent antibacterial process: VH hydrogel exhibited slightly larger inhibition zones against *S. aureus* than *MRSA*, but no significant difference was observed within the same bacterial species. Notably, the degree of VH hydrogel degradation varied with bacterial concentration, being more pronounced in high-concentration bacterial suspensions. This aligns with the degradation trend observed in Section 3.1 under different HAase concentrations. Moreover, for various concentrations of *E. faecalis*, both the inhibition zone assays of the VH-PTI composite scaffold (Fig. S2b and 2d) and those of the VH hydrogel alone (Fig. S2c and 2e) demonstrated similar trends. These results confirm that the VH-PTI composite scaffold can intelligently release antimicrobial drugs in a responsive manner, depending on the concentration of HAase produced by different bacteria.

Furthermore, we compared the antibacterial efficacy of this material with clinically used antimicrobial bone implant material PMMA. As shown in Fig. S1g–h, under simulated physiological conditions without bacterial infection (days 1–7), Van-PMMA exhibited an initial burst release of Van. However, during simulated bacterial invasion on days 11, 13, and 14 (with HAase concentration increasing alongside bacterial load), the VH-PMMA system failed to respond effectively to the infectious environment. This characteristic impedes flexible adjustment of drug release according to pathological demands, potentially leading to local subinhibitory concentrations and consequent induction of bacterial resistance [43,44]. In contrast, the VH-PTI composite scaffold functions as an intelligent release system that maintains baseline antibacterial activity under normal conditions while accelerating Van release in response to HAase during bacterial invasion, demonstrating significantly enhanced antibacterial efficacy (P < 0.001). These findings indicate that the developed VH-PTI composite scaffold possesses intelligentt drug-release characteristics, with antibacterial performance may superior to PMMA materials, highlighting its potential as an ideal infection-prevention material. Collectively, these results confirm the intelligent antibacterial capability of the VH-PTI composite scaffold, demonstrating its potential to meet the requirement for on-demand drug release and clinical application.

### 3.4 Biocompatibility and cellular behavior on the VH-PTI composite scaffold

**Fig. 6:**
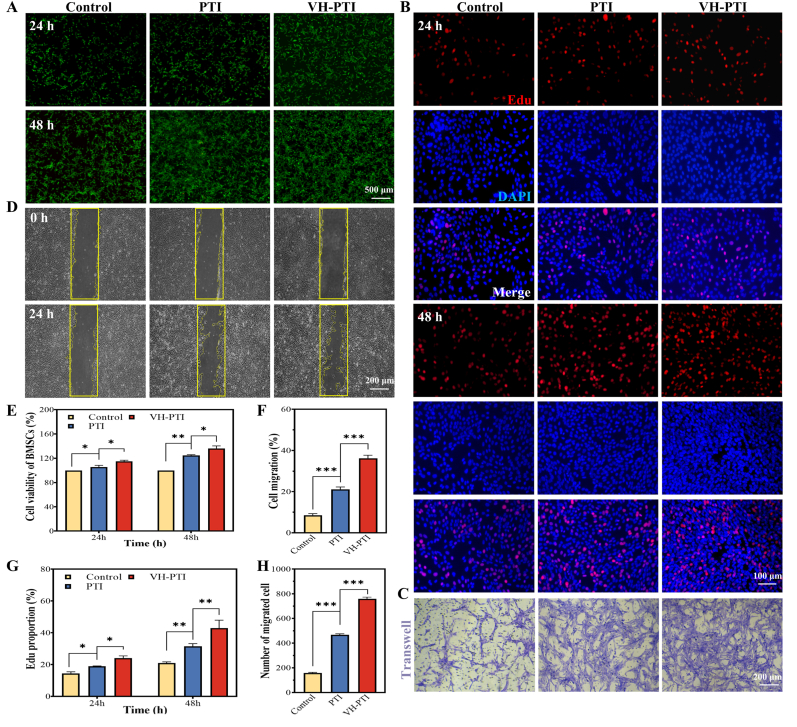
Cellular assays of scaffolds in vitro. (A–F) PTI scaffold and VH-PTI composite scaffold: (A) Live/dead and (B) Edu staining images of BMSCs co-cultured for 24 and 48 h. (C) BMSCs migration staining. (D) BMSCs scratch assay. (E) Cell viability assessed by CCK-8 assay for 24 and 48 h. Quantitative analyses: (F) scratch assay, (G) Edu and (H) migration. Data are presented as mean ± SD (n = 3,*p < 0.05,**p < 0.01,***p < 0.001).

Excellent scaffold biocompatibility remains a fundamental prerequisite for application [45]. In the hemocompatibility assessment, the hemolysis rates of the VH hydrogel, PTI scaffold, and VH-PTI composite scaffold were consistently stable at 1.0 ± 0.2 % (Fig. 3g), significantly below the international safety threshold of 5 % (p < 0.05). No significant differences were observed between groups, confirming their excellent hemocompatibility. To evaluate the cytotoxicity of the VH-PTI composite scaffold towards BMSCs, the CCK-8 assay was performed, with blank control and PTI scaffold groups serving as comparisons. As shown in Fig. 6e, cell proliferation on the PTI scaffold was significantly higher than in the blank group, highlighting its advantage. The proliferative effect was even more pronounced on the VH-PTI composite scaffold: on day 1, cell viability reached 114.3 % on the composite scaffold versus 105.7 % on PTI alone; this trend intensified by day 2, reaching 136.3 % and 124.8 %, respectively. These data indicate a synergistic enhancement in biocompatibility when combining the VH hydrogel with the PTI scaffold. Live/Dead cell staining results corroborated this conclusion (Fig. 6a). On day 1, the number of live cells (green) in the PTI group already exceeded the control, with an even more significant increase observed in the composite group, particularly evident on day 2. Similarly, EdU assays (Fig. 6b and g) confirmed that the PTI scaffold significantly enhanced cell proliferation, an effect further augmented by the addition of the VH hydrogel.

BMSCs play a pivotal role in bone repair processes due to their multipotent differentiation potential and self-renewal capacity [46]. To investigate the chemotactic response of BMSCs to the VH-PTI composite scaffold, transwell and scratch wound assays were performed. Transwell assay results (Fig. 6c) showed that after 24 h of culture, the number of migrated cells was significantly higher in both the PTI and VH-PTI composite groups compared to the blank control, with the composite group exhibiting the highest migration. Quantitative analysis of migrated cells (Fig. 6h) confirmed significant differences between the composite group, PTI group, and blank control (P < 0.001), indicating the important role of both the PTI scaffold and VH hydrogel in promoting cell migration. The scratch wound assay further validated this finding: after 24 h, the wound area was significantly reduced in the PTI and VH-PTI composite groups compared to control, with the smallest area observed in the composite group (Fig. 6d). Calculation of the migrated area (Fig. 6f) revealed a significant increase for all scaffold groups (blank, PTI, VH-PTI; P < 0.001).

### 3.5 In vitro osteogenic differentiation potential of the VH-PTI composite scaffold

**Fig. 7:**
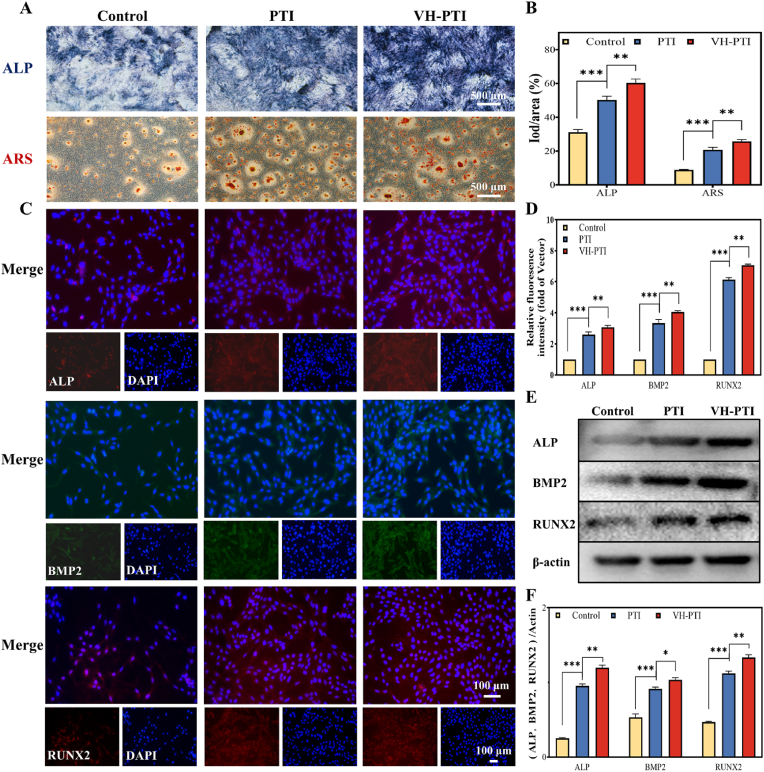
Studies on osteogenic differentiation in vitro. (A) ALP staining images on day 7 and ARS staining images on day 14 of PTI scaffolds and VH-PTI composite scaffolds, with (B) quantitative analysis. (C) Immunofluorescence staining images of osteogenesis-related proteins (ALP, BMP2, RUNX2) in BMSCs co-cultured with scaffolds for 120 h, with (D) quantitative analysis. (E) Western blot analysis of osteogenesis-related protein expression and (F) quantitative analysis. Data are presented as mean ± SD (n = 3,*p < 0.05,**p < 0.01,***p < 0.001).

Alkaline phosphatase (ALP) activity and Alizarin Red S (ARS) staining are well-established markers of osteogenic activity and typical products during osteogenic differentiation [47]. Co-culture experiments with BMSCs revealed significantly enhanced osteogenic potential in scaffold-treated groups. As shown in Fig. 7a, compared to the PTI scaffold and control, the VH-PTI composite scaffold induced a notably larger area of ALP-positive staining (blue) and denser ARS-stained mineralization nodules (red). Quantitative analysis of ALP and ARS staining (Fig. 7b) further confirmed these results, demonstrating superior osteogenic efficacy of the VH-PTI composite scaffold over the PTI scaffold.

**Fig. 8:**
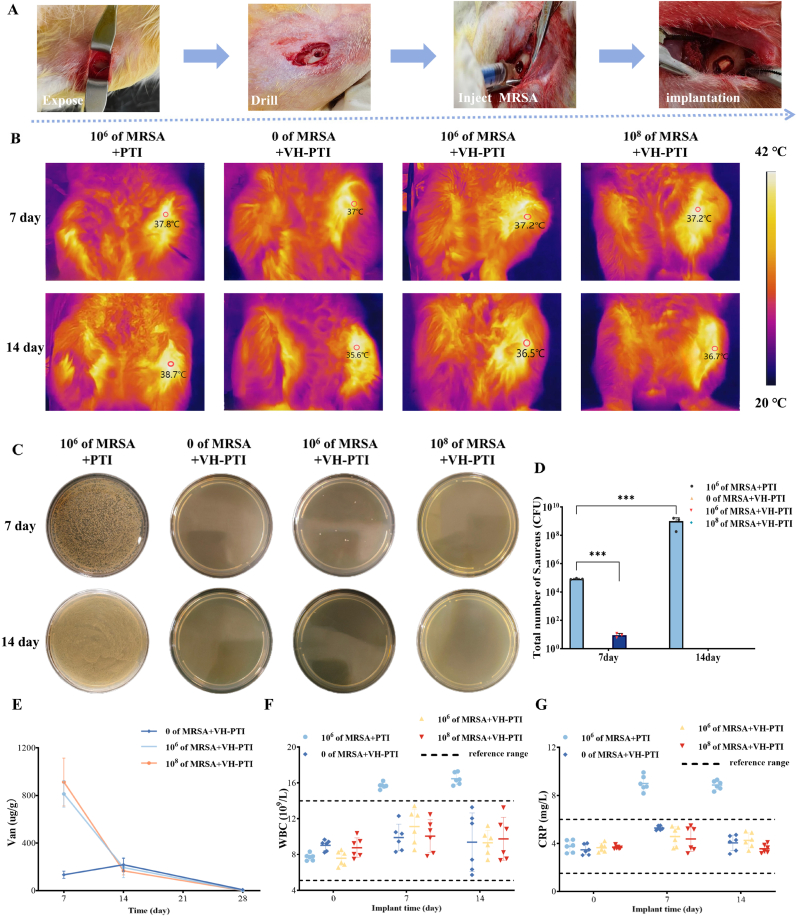
In vivo anti-infective efficacy in rabbits. (A) Schematic of the surgical procedure for establishing the rabbit tibial tuberosity infection model and scaffold implantation. The process involves the creation of a critical-sized bone defect, subsequent bacterial inoculation (for infection groups), and the precise implantation of the sterile VH-PTI composite scaffold into the defect site. (B) Infrared thermal changes at the surgical site in rabbits from each group at 7 and 14 days post-surgery. (C) Bacterial colony counts on agar plates from the surgical site in rabbits from each group at 7 and 14 days post-surgery and (D) quantitative analysis (n = 3). (E) Van release in rabbit bone tissue of the VH-PTI composite scaffold group under different infection conditions at 7, 14, and 28 days post-surgery (n = 3). (F) WBC and (G) CRP levels in rabbit serum before modeling and at 7 and 14 days post-surgery (n = 6). Data are presented as mean ± SD (***p < 0.001).

To gain deeper insight into the osteogenic promotion by the VH-PTI composite scaffold, the expression levels of key osteogenic-related proteins were examined. IF staining verified the expression of BMP-2, ALP, and Runx2. ALP is an enzyme crucial for bone growth and regeneration, serving as a key early biomarker for bone regeneration and metabolism [48]. Runx2 is a specific transcription factor that induces osteoblast differentiation and maturation, while BMP-2 is one of the most critical growth factors in this process [49,50]. Therefore, these three proteins were selected for IF staining and Western blot analysis to assess the scaffold's osteogenic capacity. As shown in Fig. 7c–d, in the VH-PTI group, Cy3-labeled ALP (cytoplasmic) and Runx2 (nuclear) exhibited the most intense red fluorescence (P < 0.01), while FITC-labeled BMP-2 (cytoplasmic) showed the brightest green fluorescence (P < 0.01), followed by the PTI group and control. Consistently, Western blot analysis (Fig. 7e–f) revealed similar protein expression trends. These results confirm that the VH-PTI composite scaffold supports BMSCs proliferation and migration while upregulating the expression of osteogenic proteins to promote in vitro osteogenic differentiation. Notably, the addition of the VH hydrogel to the VH-PTI composite scaffold significantly enhanced these effects compared to the PTI scaffold alone.

The BMP/SMAD signaling pathway is a highly conserved and functionally critical signal transduction pathway in bone formation. BMPs activate SMAD1-containing complexes and upregulate key osteogenic transcription factors such as *Runx2*, directly driving the expression of osteogenic genes. This pathway represents an important target in bone tissue engineering and skeletal disease research [51]. Additionally, the Wnt/β-catenin signaling pathway stabilizes β-catenin and activates osteogenic gene programs, serving as a key positive regulatory hub for osteoblastogenesis and bone formation [52]. Meanwhile, the MAPK signaling pathway forms a sophisticated regulatory network that integrates various extracellular signals to coordinate osteogenesis. Through crosstalk with BMP and Wnt pathways, it ensures that osteogenic activities occur at the right time, location, and intensity, functioning as a core regulatory hub in bone formation and regeneration [53]. Other pathways closely associated with osteogenesis include Hedgehog [54], PI3K/AKT [55], and NOTCH [56] signaling. To further explore the potential osteogenic mechanism of the VH-PTI composite scaffold, we extracted total RNA from cells in each group and detected the mRNA expression levels of key genes in these signaling pathways using RT-qPCR. As shown in Fig. S3a, significant changes were observed in the major genes related to the BMP/SMAD, Wnt/β-catenin, and MAPK signaling pathways, whereas genes associated with the Hedgehog, PI3K/AKT, and NOTCH pathways showed no notable alterations. Subsequent WB analysis further validated the BMP/SMAD, Wnt/β-catenin, and MAPK signaling pathways. As demonstrated in Fig. S3b–d, proteins associated with these three pathways were highly expressed, suggesting that the VH-PTI composite scaffold promotes osteogenic differentiation of BMSCs potentially through the intertwined and collective effects of these three pathways.

Based on our experimental findings, the pro-osteogenic effect of the VH-PTI composite scaffold appears to be mediated through the coordinated activation of three major signaling pathways: BMP/SMAD, Wnt/β-catenin, and MAPK. The significant upregulation of key genes and proteins associated with these pathways, as detected by RT-qPCR and Western blot analysis, strongly suggests their collective involvement. This multi-pathway activation likely creates a synergistic signaling network that efficiently drives the osteogenic differentiation of BMSCs. The BMP/SMAD pathway acts as a primary inducer of osteoblast lineage commitment, the Wnt/β-catenin pathway reinforces osteogenic specification and cell proliferation, while the MAPK pathway integrates various stimuli and fine-tunes the differentiation process through extensive crosstalk. The absence of significant changes in other pathways, such as Hedgehog, PI3K/AKT, and NOTCH, further underscores the relative specificity of this signaling signature for the VH-PTI composite scaffold.

### 3.6 In vivo evaluation of the VH-PTI composite scaffold

#### 3.6.1 Evaluation of anti-infection efficacy

**Fig. 9:**
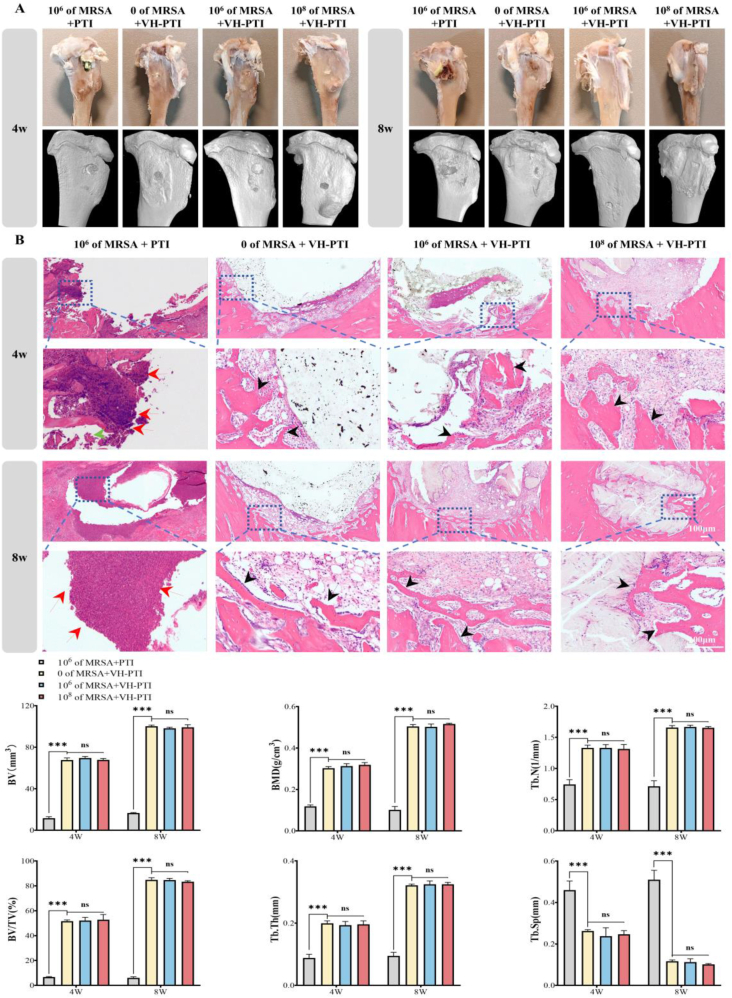
Osteogenesis and staining analysis in the rabbit tibial tuberosity defect model. (A) Gross and 3D reconstructed images of the tibia at 4 and 8 weeks post-treatment. (B) H&E staining of bone at the implant site at 4 and 8 weeks post-treatment. Arrows: green: bone chips, red: inflammatory cells, and black: new bone tissue. (C) Quantitative analysis of micro-CT parameters (BV, BV/TV, Tb.Sp, BMD, Tb.Th and Tb.N) of regenerated bone in the defect area. Data are presented as the mean ± SD (n = 3, ***p < 0.001).

To assess the preventive and intelligent antibacterial performance of the VH-PTI composite scaffold, a dynamic infection model with varying bacterial loads (0, 10^6^, 10^8^ CFU/mL) was established in rabbits, using the PTI scaffold as a control. By postoperative day 7, the control group exhibited obvious signs of infection, including visible white and yellow pus (Fig. S6). As shown in Fig. 8, Fig. 7 days after inoculation with different *MRSA* concentrations, the surgical site temperature in the control group rose to 37.8 °C, while temperatures in the VH-PTI groups were 37.2 °C, 37.2 °C, and 37.0 °C (corresponding to 0, 10^6^, 10^8^ CFU/mL groups, respectively). By day 14, only the control group temperature increased further to 38.7 °C, while the other groups remained below 37 °C. Fig. 8f–g shows elevated WBC count and CRP levels in the control group at 7 and 14 days post-infection. In contrast, WBC and CRP levels remained stable in all three VH-PTI composite scaffold groups, showing significant differences compared to the control (P < 0.01). Furthermore, body weight measurements over 8 weeks (Fig. S7) displayed a similar trend. Concurrently, bone marrow homogenates from the implant sites of each group were cultured on agar plates for 7 and 14 days to observe bacterial infection. Results (Fig. 8c–d) showed the highest *MRSA* count in the control group at day 7. In the VH-PTI composite scaffold groups, only the group inoculated with 10^6^ CFU/mL showed a small number of bacteria. By day 14, the *MRSA* count in the control group increased significantly (P < 0.001), while no bacteria were detected in the other groups. H&E staining of bone tissue further confirmed the anti-infection efficacy (Fig. 9b). At 4 weeks post-surgery, the PTI group exhibited severe inflammatory cell infiltration, which worsened significantly by week 8, confirming successful establishment of the rabbit infection model. However, no inflammatory cells were observed in any of the three VH-PTI composite scaffold groups at either 4 or 8 weeks post-surgery. These results demonstrate that the PTI scaffold lacks antibacterial properties and fails to prevent infection, while the VH-PTI composite scaffold effectively meets the demand for infection prevention and treatment in a dynamic in vivo environment.

#### 3.6.2 Evaluation of drug release in bone tissue

Rabbits from the experimental groups were euthanized at postoperative days 7, 14, and 28, and bone tissue was harvested for drug concentration measurement. As shown in Fig. 8e, at day 7, the average Van concentrations in rabbit bone tissue from the three groups (implanted with 0 CFU/mL, 10^6^ CFU/mL, and 10^8^ CFU/mL *MRSA*) were 132.16, 816.3, and 916.24 μg/g, respectively. By day 14, these concentrations decreased to 216.63, 194.43, and 164.96 μg/g, respectively. By day 28, only trace amounts of Van (7.02 μg/g) remained in the first group (0 CFU/mL), was calculated as 1.404 μg/mL based on the measured concentration ratio, which still exceeded the determined MIC (1 μg/mL) but remained below the MBEC (5 μg/mL) for the MRSA strain (Fig. S1f). These results indicate that the VH-PTI composite scaffold dynamically regulates Van release based on bacterial load, maintaining therapeutic drug concentrations at the local infection site for up to 28 days while providing sustained antibacterial protection in non-infected scenarios. This time-dependent pharmacokinetic profile validates the dual functionality of the composite scaffold: 1) rapid burst release for acute infection killing, and 2) long-term sub-therapeutic maintenance for infection prevention.

#### 3.6.3 Evaluation of bone repair capacity

Macroscopic observation and 3D reconstruction of tibiae (Fig. 9a) revealed that the tibial tuberosity defect in the PTI scaffold group progressively enlarged over time, with severe bone loss evident at both 4 and 8 weeks post-treatment. In contrast, the VH-PTI group showed a healing trend in the defect area, characterized by increased bone volume and a smoother tibial plateau surface. Assessment of bone tissue repair via H&E staining (Fig. 9b) found that at 4 weeks, the PTI group still exhibited residual bone fragments and sparse trabecular bone formation; by week 8, trabecular bone further diminished. Conversely, the VH-PTI group displayed significantly more newly formed bone tissue and trabeculae. Over time, the new bone tissue gradually thickened, its structure became more defined with increased integration at the interface with normal bone, ultimately forming a dense bone structure. Additionally, to more objectively evaluate bone repair outcomes, we conducted a quantitative analysis of the micro-CT results. As shown in Fig. 9c, in the VH-PTI three group, BV, BV/TV, BMD, Tb.Th, and Tb.N all showed an increasing trend at 4 and 8 weeks post-surgery, while Tb.Sp showed a decreasing trend. As expected, the differences between the PTI group and the VH-PTI group were statistically significant (***p < 0.001). These results confirm the superior bone repair capacity of the VH-PTI composite scaffold and its effectiveness in inhibiting *MRSA*-induced bone destruction.

#### 3.6.4 Biocompatibility assessment

**Fig. 10:**
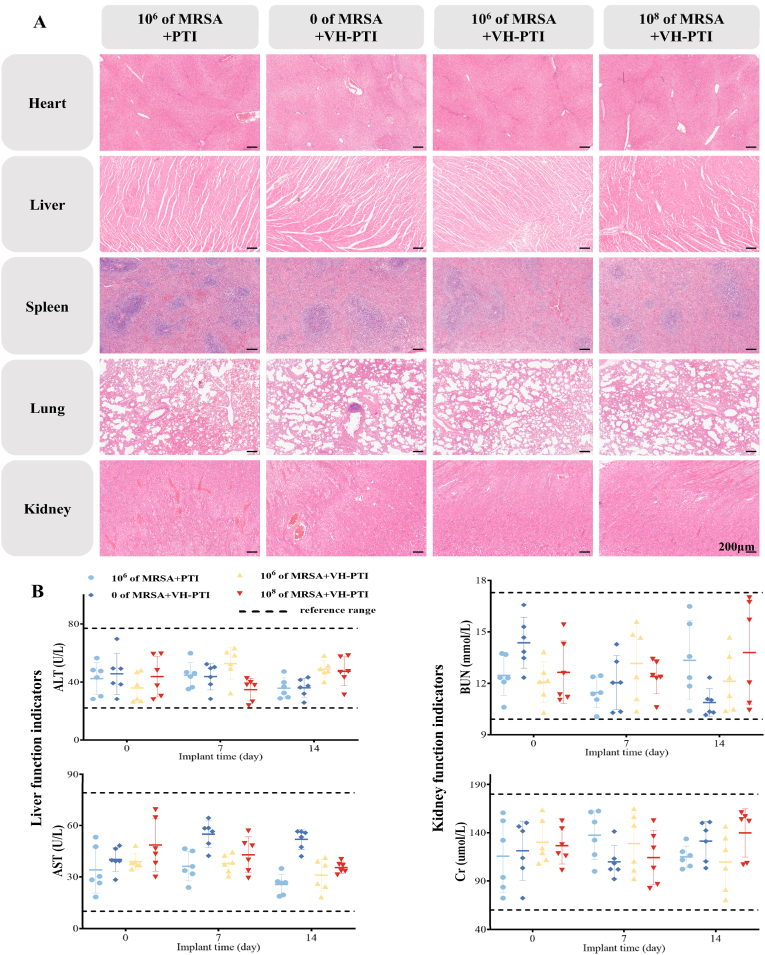
In vivo biocompatibility analysis. (A) H&E staining of major organs (heart, liver, spleen, lung, and kidney) at week 8. (B) Preoperative and postoperative (days 7 and 14) serum levels of alanine aminotransferase (ALT), aspartate aminotransferase (AST), blood urea nitrogen (BUN), and creatinine (Cr) in rabbits. n = 6, all results were within normal reference ranges. Data are presented as mean ± SD (n = 6).

Liver and kidney function tests, including alanine aminotransferase (ALT), aspartate aminotransferase (AST), blood urea nitrogen (BUN), and creatinine (Cr), were performed on rabbits before modeling and at postoperative days 7 and 14. Additionally, H&E staining of major organs (heart, liver, spleen, lungs and kidneys) was conducted at 4 and 8 weeks post-surgery to further evaluate the in vivo biocompatibility of the scaffolds. Results showed no pathological changes in the major organs of the experimental rabbits (Fig. 10a), and all serum indices remained within normal reference ranges (Fig. 10b). These results indicate that both the PTI and VH-PTI composite scaffolds exhibit excellent biocompatibility in vivo.

This study confirms the significant infection prevention and osteogenic properties of the VH-PTI composite scaffold within 8 weeks. However, due to animal ethics constraints and experimental timelines, the present study was limited to an 8-week observation period. Future studies should extend the experimental period to verify the long-term effects of the scaffold, further elucidate the synergistic interactions among mechanical support, intelligent drug release, and bone regeneration promotion, and examine potential immunogenicity risks.

## 4 Conclusion

In summary, we successfully developed an enzyme-responsive VH-PTI composite scaffold. Through its unique beehive-inspired bionic structure and functional modifications, this scaffold achieves dual optimization of intelligent antibacterial action and bone repair. Under the influence of the VH hydrogel, the composite scaffold not only retains the mechanical strength and safety profile of the original PTI scaffold but also significantly enhances BMSCs proliferation, migration, and osteogenic differentiation potential. Crucially, it enables the dynamic modulation of Van release rates in response to changes in local HAase concentration within the bacterial infection microenvironment, thereby achieving intelligent antibacterial efficacy. In a rabbit tibial defect infection model, this composite scaffold effectively prevented bacterial infection and promoted bone defect repair. Furthermore, the scaffold we constructed features a simple and safe manufacturing process, indicating potential for large-scale production. Collectively, this intelligent enzyme-responsive antibacterial scaffold holds promise as an ideal therapeutic strategy for preventing infection of bone implants and promoting bone regeneration. Future studies that expand the range of animal models and incorporate other bacterial species with different enzyme secretion profiles into the research framework could further enhance the potential for clinical translation.

## CRediT authorship contribution statement

**Jiahao Fu:** Writing – original draft, Software, Methodology, Formal analysis, Data curation. **Hai Lan:** Writing – original draft, Software, Formal analysis. **Likun Liu:** Methodology, Investigation. **Lingling Zou:** Investigation, Funding acquisition, Data curation. **Xiang Huan:** Methodology, Investigation, Funding acquisition. **Chenglin Liu:** Software, Resources. **Yaokun Wu:** Investigation, Funding acquisition. **Hao Chen:** Project administration, Methodology. **Hongzhong Xi:** Validation, Software. **Yixuan Huang:** Visualization, Funding acquisition, Formal analysis, Conceptualization. **Xiaohong Jiang:** Validation, Supervision, Project administration. **Guangquan Sun:** Writing – review & editing, Supervision, Software, Project administration. **Xin Liu:** Writing – review & editing, Visualization, Supervision. **Dong Li:** Writing – review & editing, Resources, Investigation, Funding acquisition.

## Funding

This work was supported by 10.13039/501100001809National Natural Science Foundation of China (82474342), 10.13039/501100001809National Natural Science Foundation of China (82374164), China Youth Science And Technology Talent Lifting Project (2024-QNRC2-B18), 10.13039/501100002949Jiangsu Province Youth Science And Technology Talent Lifting Project (JSTJ-2024-098), Jiangsu Provincial High-level Talent Training Program ("333 Project"): (2024) 3–2222 and Jiangsu Graduate Student Research Innovation Program(KYCX25_2223).

## Declaration of competing interest

The authors declare that they have no known competing financial interests or personal relationships that could have appeared to influence the work reported in this paper.

## Data Availability

Data will be made available on request.
