# Supplementary material for: Bioinspired honeycomb-mimetic adaptive hyaluronic acid hydrogel composite scaffold: intelligent prevention and treatment of infection of bone implants and promotion of bone repair
Source: Mater Today Bio. 2025 Nov 14;35:102558. doi: 10.1016/j.mtbio.2025.102558 (PMC12664479; doi:10.1016/j.mtbio.2025.102558)
Supplement: Multimedia component 1 [file mmc1.docx]

| **Bioinspired honeycomb-mimetic adaptive hyaluronic acid hydrogel composite scaffold: intelligent prevention and treatment of infection of bone implants and promotion of bone repair**  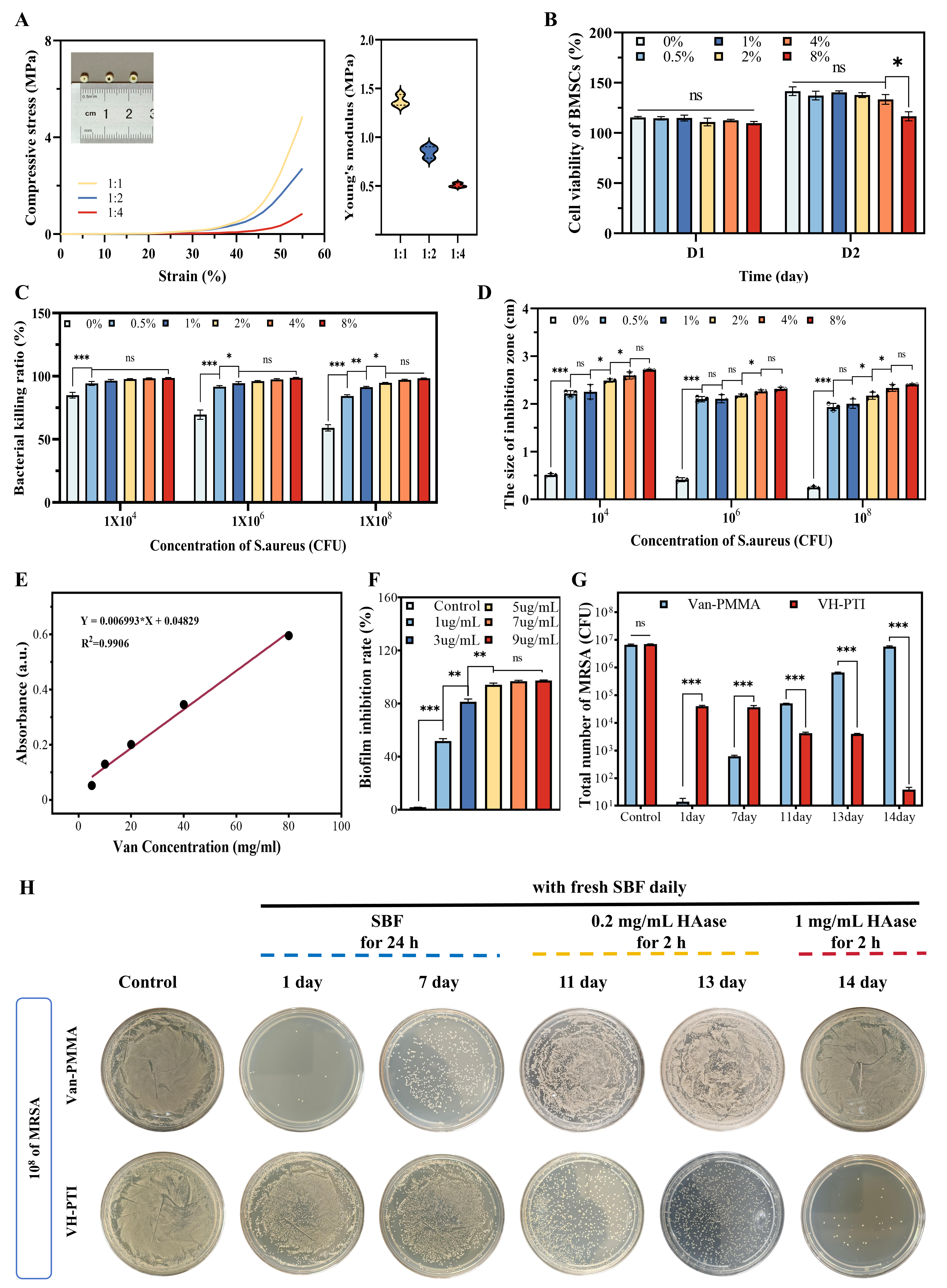 |
| --- |

| Figure S1. (A) Compressive stress tests on PTI scaffolds with outer-to-inner diameter ratios of 1:1, 1:2, and 1:4, with Young’s modulus measured. (B) Cell viability after co-culture of VH hydrogels at different concentrations with BMSCs for 1 and 2 days, assessed by CCK-8 assay. (C) Inhibition rate and (D) zone of inhibition diameter after co-culture of VH hydrogels at different concentrations with *S. aureus* at different concentrations. (E) Standard curve for Van release. (F) Biofilm inhibition rate of *MRSA* by Van at different concentrations. (H) Colony-forming unit assay and (G) quantitative results of VH-PTI composite scaffolds and Van-loaded PMMA under different intervention conditions. Data are presented as mean±SD (n = 3，*p < 0.05，**p < 0.01，***p < 0.001).   \| 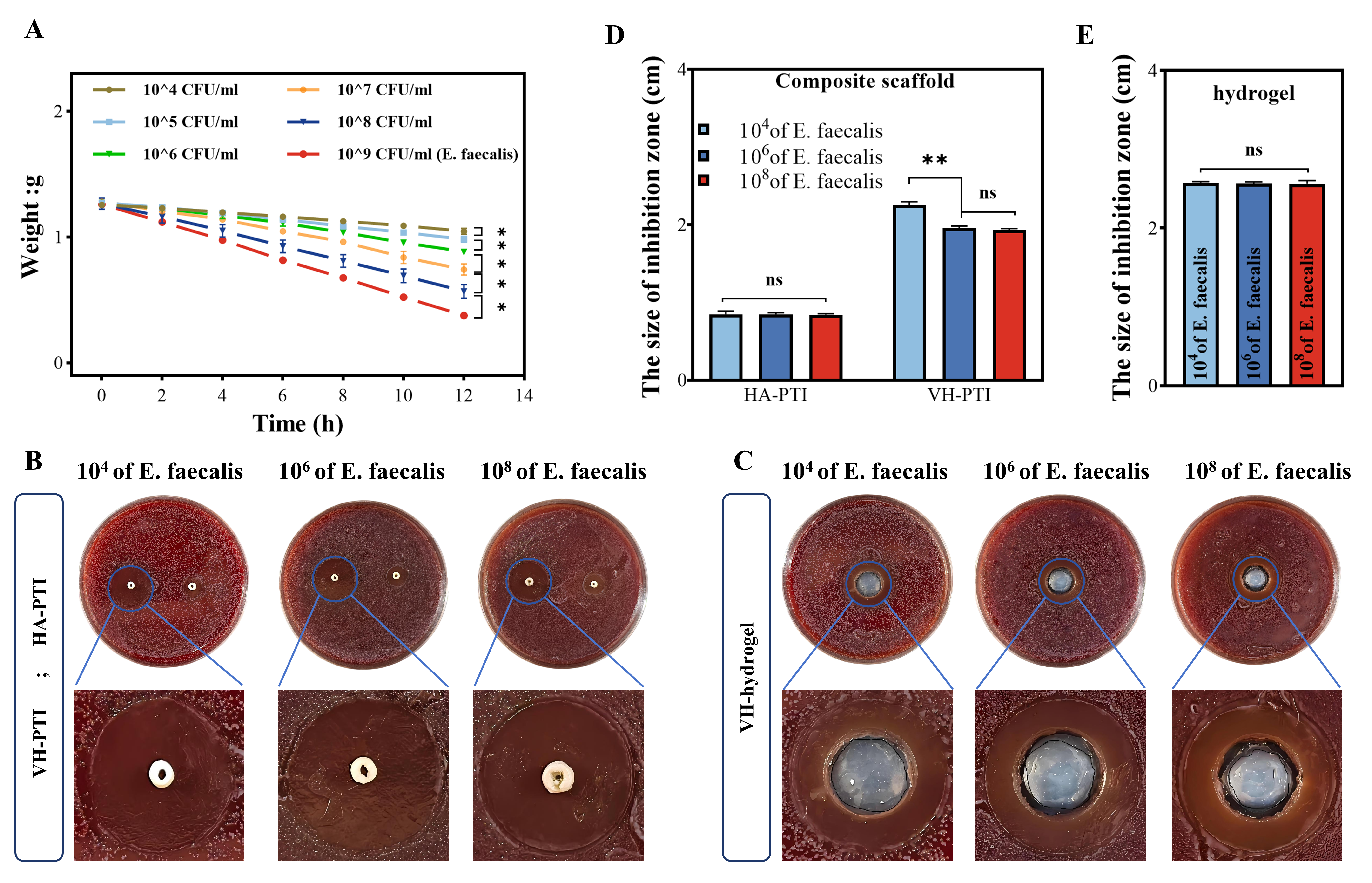 \| \| --- \| \| Figure S2. In vitro antibacterial efficacy against *E. faecalis*. (A) Mass change of VH hydrogel after 12 hours of co-culture with *E. faecalis* at different concentrations (n = 3). (B) Zone of inhibition assays of HA-PTI and VH-PTI composite scaffolds against *E. faecalis* at various concentrations and (D) quantitative analysis. (C) Zone of inhibition assays of VH hydrogels against different concentrations of *E. faecalis* and (E) quantitative analysis. Data are presented as mean±SD (n = 3，*p < 0.05，**p < 0.01). \|  \| 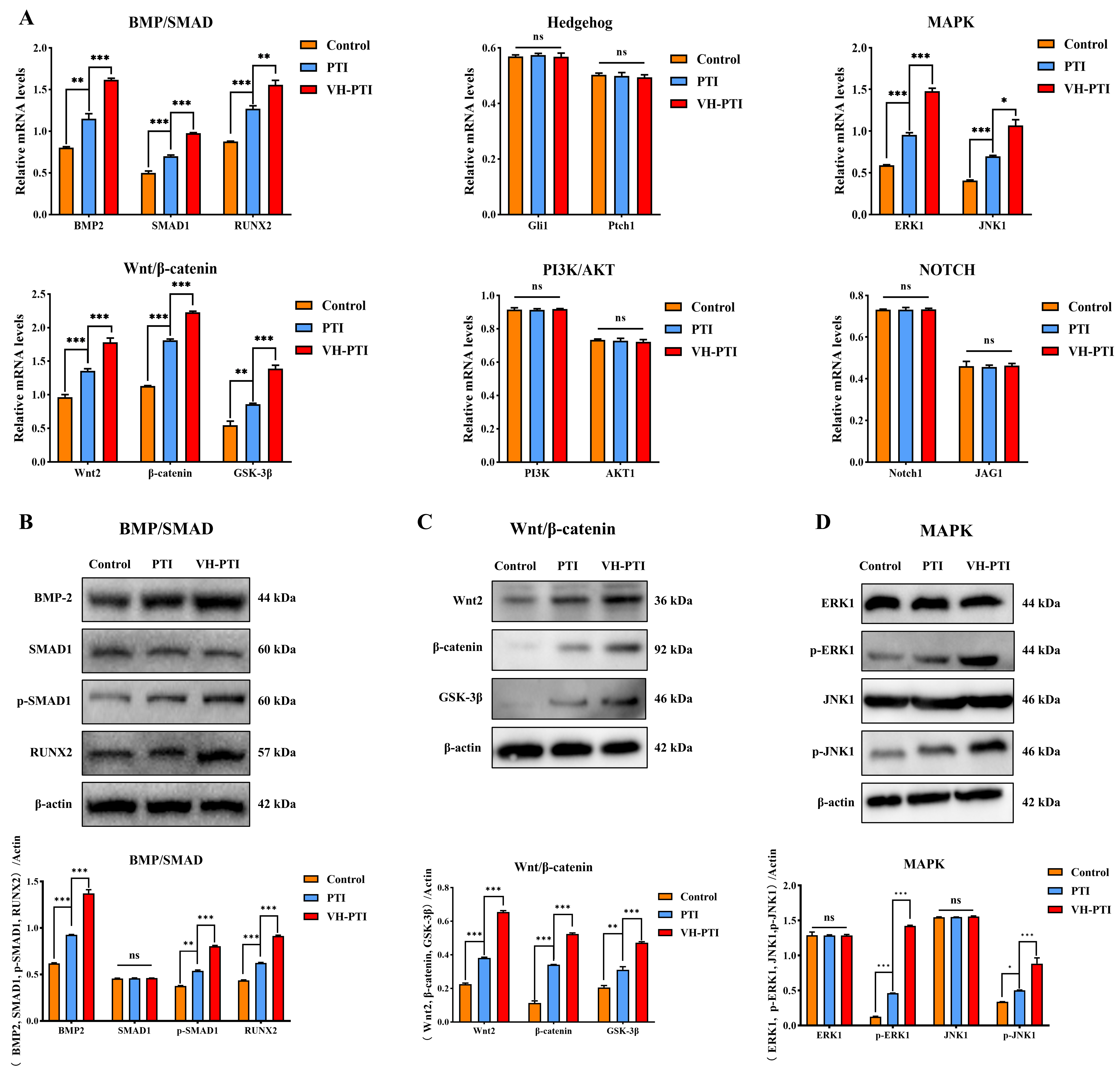 \| \| \| \| \| --- \| --- \| --- \| --- \| \| Figure S3. Studies on the osteogenic mechanism in vitro. (A) mRNA expression levels of key genes in major osteogenic signaling pathways in different groups of cells (n=6). Representative Western blot images and corresponding quantitative analysis of the (B) BMP/SMAD, (C) Wnt/β-catenin, and (D) MAPK signaling pathways in each group (n=3). Data are presented as mean±SD (*p < 0.05，**p < 0.01，***p < 0.001). \| \| \| \| \| Primer name \| Forward primer (5’-3’) \| Reverse primer (5’-3’) \| \| BMP2 \| TAGCAGGTCTTTGCACCCAG \| CCCTTCCCATCGTGACCAAA \| \| Smad1 \| CTTTCTTCCCTCCCACCACC \| ACCCTCCTCCCATTCCCAG \| \| Runx2 \| GATTCAGAGCCCAGCACCTT \| GAAACGTGTGCAAGTGACCC \| \| β-catenin \| CGCCATTTTAAGCCTCTCGG \| TGGGAAAGGTTGTGCAAGGT \| \| Wnt2 \| CCTCCAGGGTGATGTGTGAC \| CCAGTGCCATCCTGGTTCAT \| \| GSK-3β \| AAGTGAAAAGCCAAGAGGACGA \| TCCCTTGTTGGTGTTCCCAG \| \| Gli1 \| GCAGAAGGACTGTCTGGTC \| TGAGGGTGGTCAGAGTAACG \| \| Ptch1 \| AGACGACGCCGGAGAAAAAG \| CTGGGTCTGCTGGATTGAGG \| \| Notch1 \| TATCAACGGCTGCAAGAGCA \| CAGTTCTTCCAGGGGTCGTC \| \| JAG1 \| TGTGAGGCCAAACCTTGTGT \| CTGACTCTTGCACTTCCCGT \| \| PI3K \| ACCTGATGGTCTCAAAAGATGGT \| AGCTCCACCCATTGCTTCAA \| \| Akt1 \| GAGGAGATGGAGGTGTCCCT \| GTCCACGCACTCCATGCT \| \| ERK1 \| TGGAGATAGTTGGCGAGGGA \| TCTGGGTGCGGATCACTCTA \| \| JNK1 \| AGTTGGGTGCATCATGGGAG \| CACCTAAAGGAGAGGGCTGC \| \| β-actin \| GAGCGCAGCAGAAACCAGA \| CCATACCGATGAAGGAGGGC \|   Table S4. Primer sequences for RT-qPCR.   \| Antibodies \| Ratio \| \| --- \| --- \| \| BMP2 \| 1:1000 \| \| SMAD1 \| 1:1000 \| \| p-SMAD1 \| 1:1000 \| \| RUNX2 \| 1:1000 \| \| Wnt2 \| 1:1000 \| \| β-catenin \| 1:5000 \| \| GSK-3β \| 1:2000 \| \| EPK1 \| 1:2000 \| \| p-EPK1 \| 1:2000 \| \| JNK1 \| 1:1000 \| \| p-JNK1 \| 1:1000 \| \| ALP \| 1:1000 \| \| RUNX2 \| 1:1000 \| \| β-actin \| 1:10000 \| \|  \|  \|   Table S5. Primary antibodies against. |
| --- | --- | --- | --- | --- | --- | --- | --- | --- | --- | --- | --- | --- | --- | --- | --- | --- | --- | --- | --- | --- | --- | --- | --- | --- | --- | --- | --- | --- | --- | --- | --- | --- | --- | --- | --- | --- | --- | --- | --- | --- | --- | --- | --- | --- | --- | --- | --- | --- | --- | --- | --- | --- | --- | --- | --- | --- | --- | --- | --- | --- | --- | --- | --- | --- | --- | --- | --- | --- | --- | --- | --- | --- | --- | --- | --- | --- | --- | --- | --- | --- | --- | --- | --- | --- | --- | --- | --- | --- | --- | --- |
| 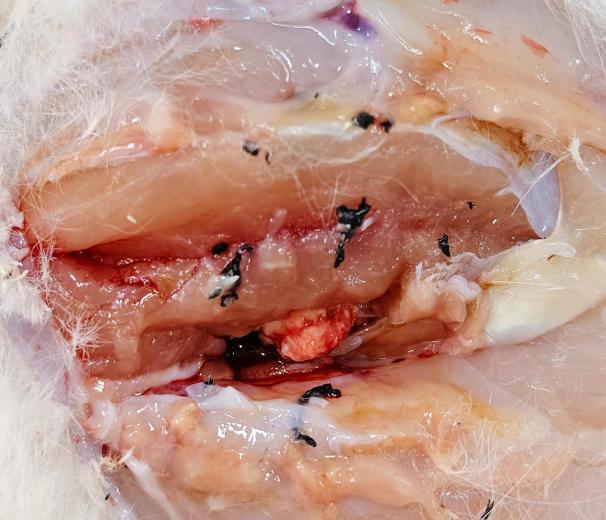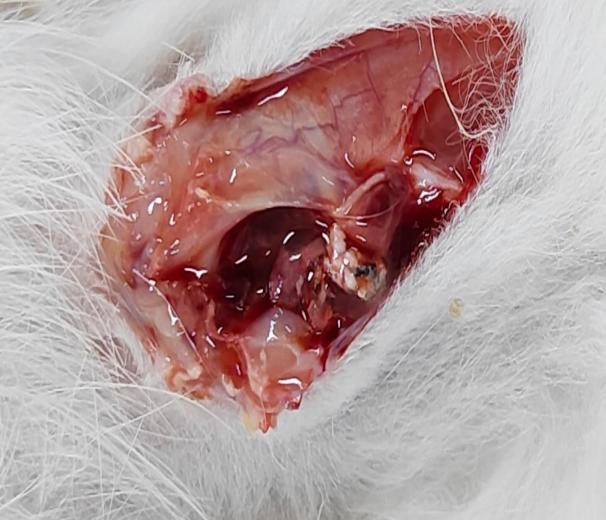 |
| Figure S6. Image of implant site infection in the control group on day 7 after surgery. |
| 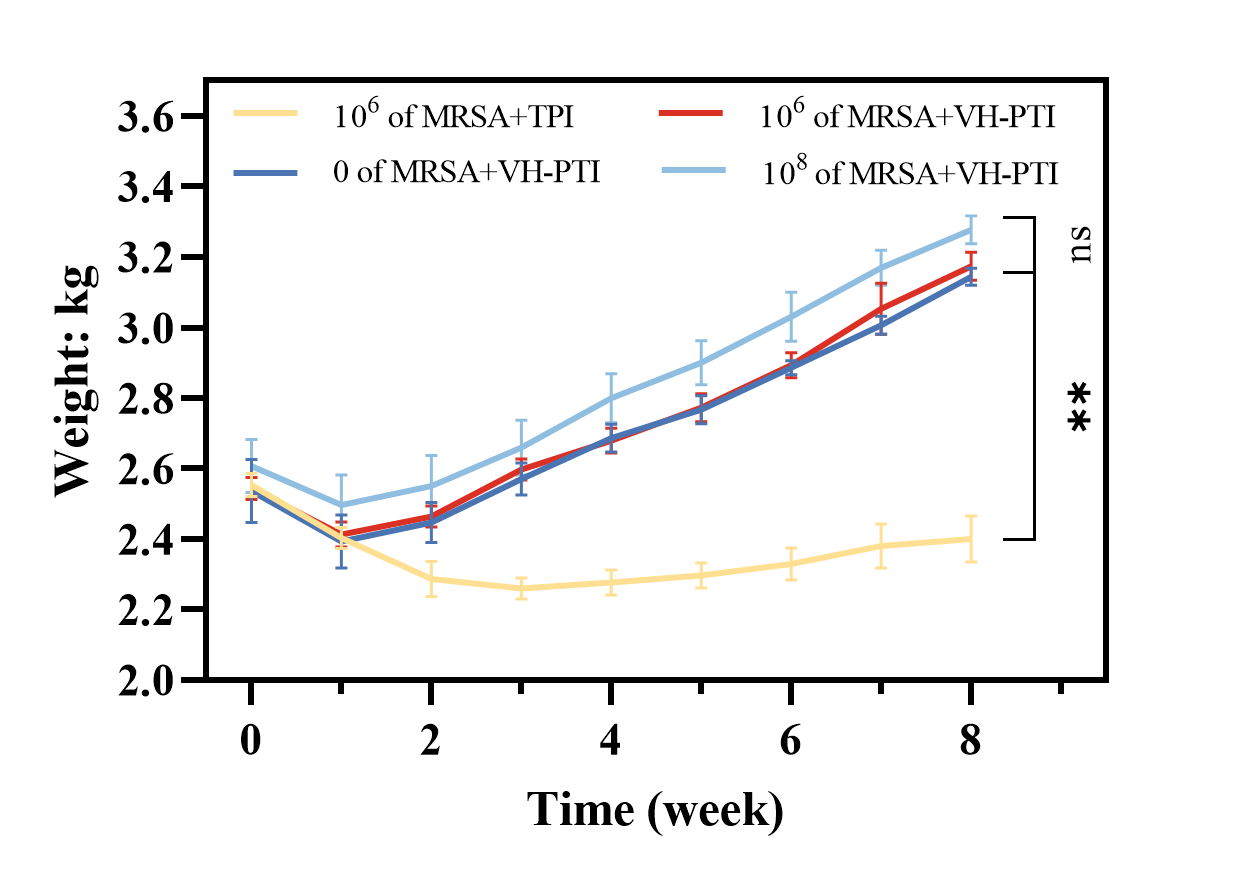 |
| Figure S7. Changes in body weight of rabbits in each group eight weeks after surgery. Data are presented as mean±SD (n = 3，**p < 0.01). |
|  |
|  |
|  |
